# Flavoromics Analysis Reveals Sex- And Size-Dependent Volatile Flavor Signatures in *Cherax quadricarinatus* Muscle Under Identical Farming Conditions

**DOI:** 10.3390/foods15203671

**Published:** 2026-10-15

**Authors:** Wen-Feng Li, An-Qi Zhao, Guang-Yu Jiang, Yi-Fei Wang, Yan Chen, Yi-Hui Wang, Lu Zhang, Chao-Chao Fu, Bing-Bing Fu, Zi-Jun Yue, Zhao-Yang Yin, Zhe Qu, Shan Zhang, Hai Huang

**Affiliations:** 1 Yazhou Bay Innovation Institute, Hainan Tropical Ocean University, Sanya 572022, China; 2 Key Laboratory of Utilization and Conservation for Tropical Marine Bioresources (Hainan Tropical Ocean University), Ministry of Education; Hainan Key Laboratory for Conservation and Utilization of Tropical Marine Fishery Resources; Hainan Tropical Ocean University, Sanya 572022, China; 3 College of Fisheries and Life Science, Hainan Tropical Ocean University, Sanya 572022, China; 4 Hainan Key Laboratory of Tropical Aquatic Germplasm, Sanya Oceanographic Institution, Ocean University of China, Sanya 572024, China

**Keywords:** red-claw crayfish, volatile organic compounds, flavoromics, body size, sex difference, butanoate metabolism

## Abstract

The red-claw crayfish, *Cherax quadricarinatus*, is a commercially valuable freshwater aquaculture species, with muscle flavor representing a core trait driving consumer preference and market value. Under identical farming conditions, same-batch crayfish commonly develop marked size divergence within and between sexes, but the impacts of this variation on muscle volatile flavor profiles remain largely unexplored. Here, *C. quadricarinatus* reared under consistent environmental conditions were categorized into four groups by body weight and sex: large-size female (GUBF), large-size male (GUBM), small-size female (GUSF), and small-size male (GUSM). Headspace solid-phase microextraction–gas chromatography–mass spectrometry (HS-SPME/GC-MS) was employed to profile volatile organic compounds. Multivariate statistical analysis, relative odor activity value calculation, metabolic pathway and network analysis, and receiver operating characteristic (ROC) analysis were combined to dissect flavor variation patterns, identify key aroma compounds, reveal underlying metabolic mechanisms, and screen potential biomarkers. Proximate composition analysis showed significant size differences in crude protein, lipid, moisture, and meat yield, with no significant sex differences. One hundred thirty-six volatile metabolites across 15 chemical categories were identified in all samples. Multivariate statistical analyses revealed clear separation among the four groups, suggesting that both body size and sex shape muscle volatile metabolite profiles, with stronger effects observed in females. Thirty-eight shared aroma-active compounds were detected, mainly including alcohols, aldehydes, ketones, esters, and aromatic hydrocarbons. The overall sensory profile was dominated by sweet-like notes, followed by fruity and green notes. Sex-related flavor differences were relatively slight in large-size crayfish but pronounced in small-size individuals. Large-size female crayfish had simpler flavor composition, while small-size females showed higher diversity and abundance of flavor compounds; size variation had limited effects on male flavor. Butanoate metabolism was significantly enriched in both the small-size sex comparison and female size comparison. Core metabolic frameworks were generally conserved across groups, but key hub metabolites differed by comparison type. This study systematically characterized the volatile flavor profiles of *C. quadricarinatus* muscle across different body sizes and sexes under controlled farming conditions, providing preliminary references for quality evaluation and premium aquaculture optimization of *C. quadricarinatus*.

## 1. Introduction

*Cherax quadricarinatus*, commonly known as red-claw crayfish or Australian freshwater crayfish, belongs to the genus *Cherax*, within the family Parastacidae, order Decapoda, class Malacostraca, and phylum Arthropoda. This species is native to the river systems of northern Queensland, Australia, and Papua New Guinea. Because of its delicate flesh, desirable flavor, strong tolerance to long-distance transportation, and high survival rate for live-market sales, *C. quadricarinatus* features simple artificial propagation procedures with low technical barriers and breeding costs [1,2,3]. Given these superior cultivation characteristics, it has been widely introduced and artificially farmed in numerous countries across Asia, Europe, and Africa, emerging as a globally important freshwater crayfish species with high economic value [4]. The crayfish was first introduced to Taiwan Province of China in 1990, and farming industries were subsequently established in Anhui, Jiangsu, Zhejiang, Fujian, Guangdong, Guangxi, and Hainan provinces [5,6]. In 2024, the domestic yield of *C. quadricarinatus* in China reached 185,000 tons, with its cultivation area and output exhibiting a continuous annual growth trend. *C. quadricarinatus* possesses a tender muscle texture, pleasant taste, and a high edible ratio, with the meat rate at 18% of its total body weight, which contributes to its high consumer acceptance [7]. As the primary edible tissue of red-claw crayfish, the muscle is rich in essential amino acids and polyunsaturated fatty acids [6]. In addition, flavor is a critical determinant of consumer purchasing decisions in the seafood market and includes taste and aromatic attributes [8]. Current seafood flavor research predominantly focuses on flavor profiles in cooked muscle tissues, whereas studies targeting raw seafood muscle remain limited [8]. Although raw seafood tissues present relatively weak volatile aromatic characteristics, several core flavor-active compounds can be retained after thermal processing [9]. Accordingly, it is essential to systematically characterize the key aromatic substances in raw crayfish muscle tissues to supplement existing seafood flavor research systems.

In commercial cultivation of *C. quadricarinatus*, pronounced phenotypic growth variation is frequently observed among juveniles from the same batch with uniform initial body size, even when reared under standardized feeding regimes, consistent habitat conditions, and identical culture cycles [10]. Distinct growth disparities exist not only between male and female crayfish but also among adult individuals of the same gender [10]. Sexual size dimorphism is a well-documented biological trait in crustacean species [11,12,13]. *C. quadricarinatus* exhibits typical dimorphic growth characteristics, with male individuals generally achieving faster growth rates and larger final body size than female counterparts [14,15]. Both inter-sex and intra-sex body size discrepancies can be attributed to multiple endogenous and exogenous factors, including genetic polymorphism, individual health status, nutrient utilization efficiency, environmental fluctuation, and intestinal microbial community structure [16,17]. Despite uniform farming conditions, pronounced differences in growth rates among crayfish of different genders and body sizes may substantially alter muscle quality properties, particularly the accumulation and composition of volatile aromatic compounds. Similar growth-differentiated flavor variation has been widely reported in domesticated poultry species [8]. Long-term artificial domestication and breeding have generated fast-growing and slow-growing poultry strains with distinct flavor profiles [8]. For instance, indigenous Chinese chicken breeds contain higher concentrations of characteristic flavor substances than commercial broilers [18]. Significant differences in volatile metabolite profiles have also been identified between Taihe silky fowl (a typical slow-growing strain) and Cobb broilers, screening 24 volatile compounds and recognizing them as potential flavor biomarkers for strain differentiation [19]. By analogy with the flavor discrepancies between fast- and slow-growing poultry strains [8], the pronounced body size variation among *C. quadricarinatus* individuals under consistent culture conditions may cause considerable differences in muscle volatile aromatic characteristics across genders and body sizes. Moreover, Consumer priorities regarding aquatic products have gradually shifted from high nutritional value to superior flavor quality, a trend particularly evident for species suitable for raw consumption or with sashimi-grade production potential. In contrast to thermally processed products, where cooking-driven reactions such as the Maillard reaction and lipid degradation generate a wide array of secondary flavor compounds, the sensory attributes of raw aquatic products derive almost entirely from their endogenous volatile profiles. Without heat-induced flavor alterations, subtle differences in the composition of odor volatile compounds (OVCs) translate directly into perceivable variations in aroma profile, freshness perception, and overall palatability. As one of the most influential palatability attributes, odor accounts for approximately 80% of overall food flavor perception and strongly influences product quality and consumer acceptability [20,21]. Especially red-claw crayfish may serve as a partial substitute for other traditional raw shrimp species in raw seafood culinary practices such as Chinese raw marination or sashimi preparations. Therefore, characterizing key volatile flavor compounds in the muscle tissues of *C. quadricarinatus* across genders and body sizes (cultivated from unified seedlings and standardized farming systems) is of great practical value. The findings can support graded pricing and precision marketing of crayfish products in segmented commercial markets. Additionally, it can provide novel theoretical references for germplasm improvement, selective line breeding, and new strain cultivation of *C. quadricarinatus*.

Continuous progress in analytical techniques, particularly advances in separating, detecting, and qualitatively characterizing odor-active molecules from complex volatile mixtures, has greatly deepened understanding of flavor profiles in aquatic products. Emerging analytical tools can resolve mixed volatile organic compound (VOC) systems present in complicated food matrices [22], enabling the capture and differentiation of various VOCs dominated by aldehydes, alcohols, and sulfides. As essential constituents of food flavor fingerprints, these volatile substances endow modern instrumental detection with prominent technical merits, including high sensitivity and analytical objectivity in meat flavor evaluation. Widely adopted in food quality monitoring and assessment, metabolomic approaches facilitate the screening and identification of flavor-related metabolites across diverse food substrates [23]. Headspace solid-phase microextraction (HS-SPME) is a solvent-free, time-efficient, and user-friendly pretreatment technique for volatile compound analysis, that integrates sampling, extraction, enrichment, and injection into a unified analytical workflow [24]. The combination of headspace solid-phase microextraction (HS-SPME) and gas chromatography-mass spectrometry (GC-MS) has been proven reliable for identifying volatile substances in a broad range of food products, covering fruits [25,26], tea [27], crops [28,29,30], meat products [31], and Chinese medicinal herbs [32]. In aquatic nutrition and flavor research, this integrated analytical system has been applied to investigate multiple flavor-related topics. Existing studies have tracked dynamic variations in volatile chemicals in salmon (*Oncorhynchus* spp.) and large yellow croaker (*Pseudosciaena crocea*) during frozen storage [33,34], characterized volatile compound profiles of sea bass throughout smoking [35], and compared flavor component differences in *Chlamys farreri* samples during drying [36]. Additional applications involve volatile component profiling of Atlantic shellfish, including *Mytilus galloprovincialis*, *Venerupis pullastra*, *Ostrea edulis*, *Ensis ensis*, *Cerastoderma edule* and *Pollicipes cornucopia* [37], sensory-associated volatile analysis of fresh oysters *Crassostrea ariakensis* [38], and quantitative detection of pyridine in crustacean tissues and marine sediments [39]. Moreover, this technique supports the identification of odor-active compounds in cooked *Eriocheir sinensis* muscle [40], characterization of flavor alterations in grass carp (*Ctenopharyngodon idellustissues*) after broad bean feeding [41], and exploration of habitat-dependent differences in hepatopancreas lipids and volatile metabolites of mud crab *Scylla paramamosain* [42]. Furthermore, multivariate statistical models, such as principal component analysis (PCA) and orthogonal partial least squares discriminant analysis (OPLS-DA), can effectively distinguish flavor profiles of samples with varied processing treatments, muscle tissue types, and geographical origins. This integrated analytical strategy effectively overcomes the inherent drawbacks of traditional sensory evaluation, which is highly subjective and labor-intensive [43,44]. Despite the extensive application of gas chromatography-ion mobility spectrometry (GC–IMS) in food flavor analysis, few studies have focused on exploring the muscle flavor characteristics of red-claw crayfish.

Accordingly, the red-claw crayfish aquaculture sector in China has grown robustly in recent years, with continued expansion in production volume and farming area. Nevertheless, no standardized, industry-wide criteria currently exist to assess crayfish product quality. Sexual dimorphism in crayfish, along with growth rate variation driven by multiple biotic and abiotic factors, creates considerable heterogeneity in product quality even within a single farming batch. Therefore, a unified evaluation framework for the quality attributes and flavor profiles of market-ready crayfish across size grades and sexes is needed to support refined production management and market grading. To address this gap, the present study used juvenile crayfish with similar initial body sizes from the same cohort, reared under standardized aquaculture conditions until they reached maturity and diverged in size. We systematically characterized the volatile flavor fingerprint of *C. quadricarinatus* muscle tissue using headspace solid-phase microextraction coupled with gas chromatography–mass spectrometry (HS-SPME/GC–MS) and multivariate statistical analysis, and further identified possible key aroma-active components. The findings provide fundamental data and theoretical underpinnings for quality improvement and precision farming of cultured red-claw crayfish.

## 2. Materials and Methods

### 2.1. Experimental Animals and Culture Conditions

The rearing conditions for experimental animals *C. quadricarinatus* followed those described in our previous study [10]. In brief, 400 red-claw crayfish were collected from three adjacent culture ponds in a commercial aquaculture farm in Sanjiang, Hannan province, China. All red-claw crayfish were selected from larvae of similar sizes (4.15 ± 0.34 cm; 7.43 ± 0.21 g) in the same batch. All the red-claw crayfish juveniles were purchased from the same commercial aquaculture farm in Sanjiang and were offspring from the farm’s own captive breeding program. The three adjacent culture ponds were independent of each other and had the same floor area, rearing density, water source, and aquaculture setup. All the crayfish were fed commercial feed for 3 months under consistent farming conditions. Environmental conditions are as follows: temperature, 28–32 °C; pH, 7.4–8.0; dissolved oxygen ≥ 6.0 mg/L; ammonia nitrogen ≤ 0.20 mg/L; nitrite ≤ 0.1 mg /L. The stocking density is originally 5000 crayfish per 667 m^2^. The diet used in this study was a commercial feed purchased from Hainan Baiyang Feed Co., Ltd. (Hainan, China), with crude protein ≥38%, crude lipid ≥4.5%, crude fiber ≤5.0%, crude ash ≤15.0%. The crayfishes cultured in this study were fed twice per day: before 8:00 am and after 6:00 pm.

### 2.2. Sample Collection

Experimental animals were randomly captured using cage traps in each culture pond. All crayfish in the cages were collected, and their body weights were measured. Experimental animals were grouped as described in our previous study [10]. In brief, the crayfish with the top 10% and bottom 10% daily weight gain were assigned to the high-body-weight and low-body-weight groups, respectively. In total, 30 larger-sized females (BF; 51.35 ± 5.66 g) and larger-sized males (BM; 67.23 ± 14.46 g) were randomly selected from the high-body-weight group, respectively. At the same time, 30 smaller-sized females (SF; 25.86 ± 4.99 g) and smaller-sized males (SM; 26.37 ± 4.69 g) were randomly selected from the low-body-weight group. All crayfish were dissected using sterilized and flamed surgical tools under sterile conditions. Abdominal muscle was dissected and surface-sterilized with 75% ethanol three times, and rinsed three times in sterile phosphate-buffered saline (PBS). All muscle samples (MUBF, MUBM, MUSF, and MUSM) were snap-frozen in liquid nitrogen upon collection and stored at −80 °C until use. Muscle samples from five crayfish of the same gender and size group were randomly pooled as one composite sample. Each group contained five biological replicates.

### 2.3. Muscle Proximate Composition

The proximate composition in muscle, including crude protein, crude lipid, ash, and moisture, in MUBF, MUBM, MUSF, and MUSM groups was determined according to the standard methods of the Association of Official Analytical Chemists (AOAC, 2000) [45]. Meat yield was assessed via a standardized dissection and weighing procedure [7]. In brief, the experimental animals were first wiped with 75% (*v*/*v*) ethanol and allowed to dry thoroughly. Decapitation was carried out at the cephalothorax–abdomen carapace boundary, after which the heart, gonads, and associated viscera located at the thoracic-abdominal junction were removed, and all appendages were cut away. The telson was detached from the abdominal segment via gentle rotation, and the digestive tract was extracted. Bilateral incisions along the abdominal flanks in a ventral-to-dorsal direction were made with scissors, and the ventral exoskeletal plate was lifted off with forceps. Abdominal muscle was carefully excised with scissors and spatula; residual fibers adhering to the carapace were collected separately with forceps and pooled. Surface moisture was removed with moist gauze, and the abdominal muscle mass was recorded on an analytical balance. Muscle yield rate was subsequently calculated via the following formula:

Muscle yield (%) = Abdominal muscle weight/Whole body weight × 100.

### 2.4. Volatile Compound Analysis by HS-SPME/GC–MS

The headspace solid-phase microextraction combined with gas chromatography-mass spectrometry (HS-SPME/GC–MS) was conducted to separate and characterize volatile organic compounds (VOCs) in red-claw crayfish muscle. Muscle tissue was pulverized into a fine powder in liquid nitrogen, and exactly 100 mg of homogenized muscle powder was placed in a 20 mL headspace vial. 5 mL saturated sodium chloride solution containing a deuterated n-hexanol internal standard (10 mg/L, diluted at 100: 2, *v*/*v*), was added into the headspace vial and mixed well. Separately, 10 μL of 10 mg/L n-alkane mixed standard solution was added into another empty headspace vial for subsequent qualitative retention index calibration. All prepared samples were analyzed by GC–MS to obtain VOC fingerprint profiles.

Volatile compound extraction and instrumental analysis were conducted utilizing a solid-phase microextraction (SPME) ARROW fiber (50/30 μm, DVB/CAR/PDMS, StableFlex, 2 cm; Supelco, Bellefonte, PA, USA) mounted on an Agilent 7890B gas chromatograph coupled with an Agilent 5977B mass spectrometer (Agilent Technologies, Santa Clara, CA, USA), equipped with a DB-Wax capillary column (30 m × 250 μm × 0.25 μm; Agilent Technologies, Santa Clara, CA, USA). The programmed column temperature gradient was performed as follows: an initial hold at 40 °C for 2 min, followed by a ramp of 20 °C/min to 220 °C (zero hold time), a continuous increase of 45 °C/min to 245 °C, and a final isothermal maintenance at 245 °C for 6 min. All critical instrumental parameters were precisely set throughout the extraction and detection processes. The VOC incubation temperature was maintained at 60 °C, with 6 min preheating, 10 min extraction, and then 2 min fiber desorption. The injection port operated in splitless mode, with a septum purge flow of 5 mL/min. 99.999% helium served as the carrier gas at a constant column flow of 1 mL/min. The injection port, transfer line, and ion source temperatures were stabilized at 240 °C, 240 °C, and 300 °C, respectively.

Mass spectrum acquisition was performed in a full-scan mode at a resolution of 30,000, covering a mass range of 35–450 m/z, with a solvent delay of 2.45 min. Fiber conditioning was implemented at 250 °C, consisting of 5 min pre-desorption equilibration and 2 min post-desorption stabilization to ensure stable extraction performance.

### 2.5. Raw Data Analysis

The acquired raw mass spectral data were processed via MSDIAL software (V4.9) [46], involving peak screening, baseline correction, spectral deconvolution, peak integration, peak alignment, and compound annotation. To reduce analytical noise and systematic bias, raw data were further processed via outlier elimination, missing value filtering, missing value imputation (missing values were filled in with half of the minimum value), and total ion current normalization method was employed in this data analysis. The final dataset, containing peak number, sample name, and normalized peak area, was imported into in-house R scripts (Biotree DB3.0) for multivariate analysis. Data were scaled and logarithmic transformed to minimize the impact of both noise and high variance of the variables. Compound identification was accomplished by matching acquired spectra against a self-established high-resolution database and the NIST standard mass spectral library (NIST 23), respectively. Each treatment was tested with five biological replicates to guarantee the reliability and reproducibility of analytical results.

### 2.6. Identification of Key Vocs via Relative Odor Activity Value (Roav) Calculation

Relative Odor Activity Value (ROAV) was adopted in this study to quantitatively assess the contribution of individual volatile components to the overall flavor profile of red-claw crayfish muscle and to screen the core flavor-active compounds. The ROAV calculation procedure was performed in accordance with the analytical method established by Liu et al. (2008) [47]. Briefly, the relative percentage content (C%) of each volatile compound was calculated based on the average peak area obtained by GC–MS detection. Only volatile compounds with available odor threshold parameters were retained for subsequent flavor contribution evaluation. The compound dominating the overall flavor characteristics of crayfish muscle was defined as the standard substance, with ROAV value as 100 (ROAV_stan_ = 100). The ROAV values of all other detected volatile compounds (ROAV_A_) were then calculated based on the unified formula. A higher ROAV value indicates a more prominent flavor contribution of the corresponding compound to the sample’s overall sensory characteristics. The specific calculation formula is presented as follows:

ROAV_A_ ≈ 100 × C%_A_/C%_stan_ × T_stan_/T_A_

In this formula, C%_A_ and C%_stan_ represent the relative percentage content of the target volatile compound and the relative percentage content of the predominant flavor compound with the maximum overall flavor contribution, respectively. T_stan_ and T_A_ denote the odor threshold of the standard predominant flavor compound and the odor threshold of the target volatile compound, respectively.

Overall, the ROAV metric serves as a critical criterion for characterizing the flavor profiles of experimental samples, with its maximum theoretical value set to 100. ROAV values ≥ 1 are defined as key aroma-active compounds in the examined specimens, whereas volatile components falling within the range of 0.1 ≤ ROAV < 1 are regarded as essential modulators contributing to the holistic flavor perception of samples [48].

### 2.7. Statistical Analysis

Principal component analysis (PCA), an unsupervised analysis that reduces the dimension of the data, was carried out to visualize the distribution and the grouping of the samples. 95% confidence interval in the PCA score plot was used as the threshold to identify potential outliers in the dataset. Moreover, supervised orthogonal projections to latent structures discriminant analysis (OPLS-DA) was applied to visualize group separation and identify significantly changed metabolites. A 7-fold cross-validation was performed to calculate the value of R^2^ and Q^2^, which indicates how well the variation in a variable is explained and how well a variable could be predicted, respectively. To check the robustness and predictive ability of the OPLS-DA model, 1000 permutations were further conducted, and the R^2^Y and Q^2^ intercept values were obtained. Furthermore, value of variable importance in the projection (VIP) of the first principal component in OPLS-DA analysis was calculated. The metabolites with VIP > 1 and *p* < 0.05 (Student’s *t*-test) were considered as significantly changed metabolites. In addition, commercial databases including KEGG (http://www.genome.jp/kegg/) and MetaboAnalyst (http://www.metaboanalyst.ca/) [49] were used for pathway enrichment analysis. Additionally, the correlation network among non-volatile differential metabolites was constructed and visualized using Cytoscape_v3.9.0 to elucidate the interactive relationships among key metabolites.

## 3. Results

### 3.1. Basic Muscle Compositions

Among the experimental groups (large-sized female, BF, large-sized male, BM, small-sized female, SF, and small-sized male, SM), all *C. quadricarinatus* were reared under identical culture conditions for an equivalent rearing period. Apart from the significantly higher body weight observed in the BM group relative to the BF, SF, and SM groups, significant size-dependent variations were detected for most body-composition parameters, including crude protein, crude lipid, moisture, and meat yield, except ash content. By contrast, no significant sex-related differences were identified across these measured indices. Specifically, crude protein, crude lipid, moisture, and meat yield did not differ significantly between BF and BM (Table 1). Nevertheless, in the comparison of BF and SF, BM and SM exhibited statistically distinct values (*p* < 0.05), respectively, accompanied by a declining trend of these compositional parameters in larger-bodied individuals (Table 1).

### 3.2. Analysis of Volatile Compounds by Hs-Spme/gc–ms

**Figure 1:**
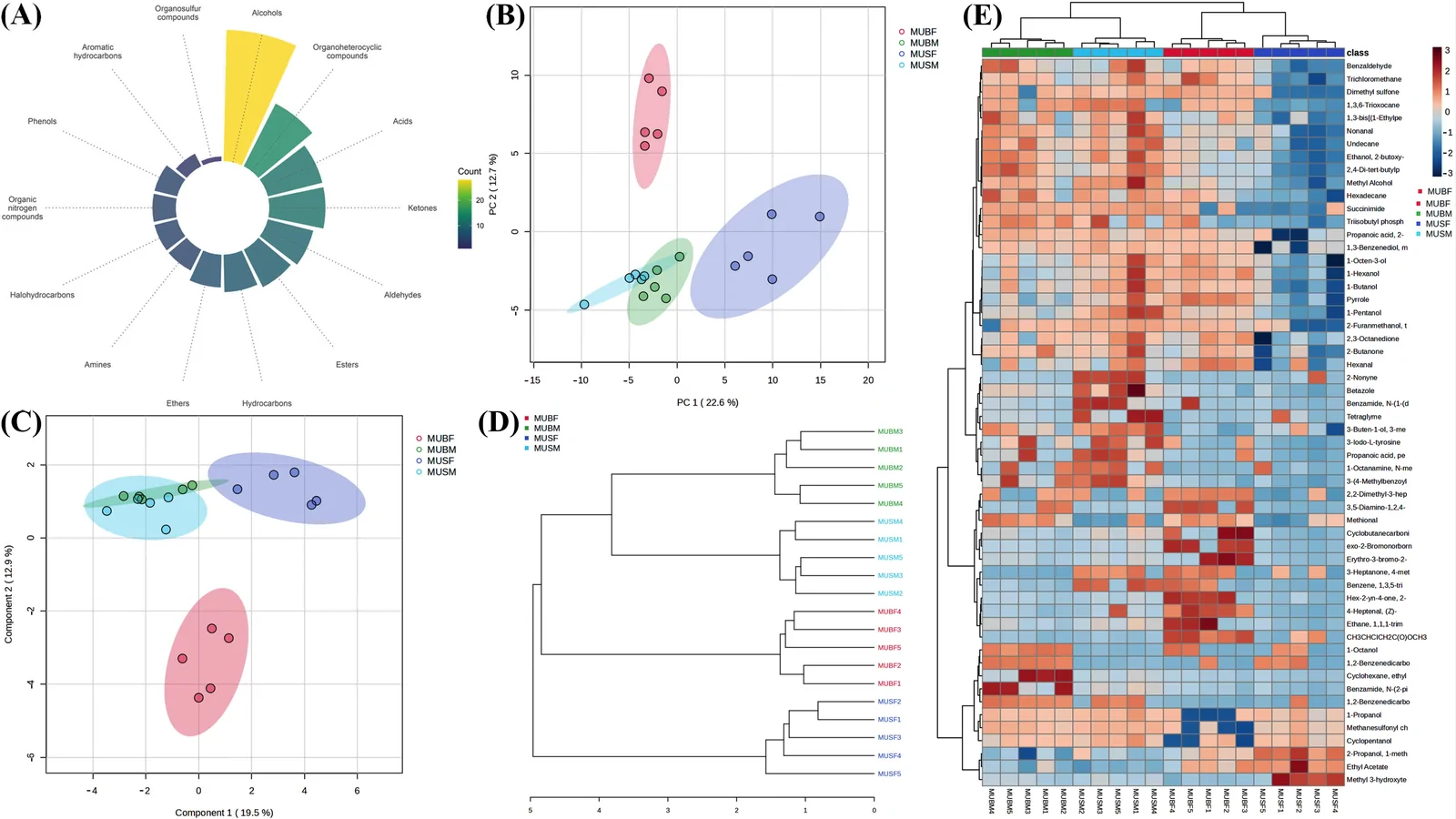
Multivariate statistical analysis of volatile compound profiles for *C. quadricarinatus* muscle samples. (**A**) Donut Plot of volatile compound classification; (**B**) PCA score plot showing the spatial distribution of MUBF, MUBM, MUSF, and MUSM sample groups. (**C**) sPLS-DA score plot presenting the distribution of MUBF, MUBM, MUSF, and MUSM sample groups. (**D**) Dendrogram plot showing clustering outcomes and the degree of inter-group differentiation across the MUBF, MUBM, MUSF, and MUSM sample groups; (**E**) Heatmap showing the clustering profiles of metabolites with significant abundance variation among the MUBF, MUBM, MUSF, and MUSM sample groups.

A total of 172 volatile metabolites were detected and preliminarily identified via HS-SPME/GC–MS analysis. Of these, 36 metabolites failed to be matched with valid chemical information in the spectral databases or could not be taxonomically classified. The remaining 136 identifiable volatile compounds were further categorized into 15 chemical classes (Figure 1A). Specifically, these compounds consisted of 28 alcohols (20.59%), 15 organoheterocyclic compounds (11.03%), 12 acids (8.82%), 12 ketones (8.82%), 10 aldehydes (7.35%), 9 esters (6.62%), 8 hydrocarbons (5.88%), 7 ethers (5.15%), 5 amines (3.68%), 5 halohydrocarbons (3.68%), 5 organic nitrogen compounds (3.68%), 5 phenols (3.68%), 3 aromatic hydrocarbons (2.21%), 1 organosulfur compound (0.74%), and 11 unclassified substances grouped as others (8.09%).

### 3.3. Multivariate Data Analysis

Principal component analysis (PCA) was applied to characterize the global divergence of volatile metabolite profiles across four experimental groups: large-sized female crayfish muscle (MUBF), large-sized male crayfish muscle (MUBM), small-sized female crayfish muscle (MUSF), and small-sized male crayfish muscle (MUSM). As shown in the two-dimensional PCA score plot (Figure 1B), the four groups showed clear separation, indicating distinct volatile compositions modulated by sex and body size. Specifically, the MUBM and MUSM groups were positioned close together in the PCA coordinate system, suggesting greater similarity in muscle volatile profiles between male crayfish of different size grades. In contrast, both female groups (MUBF and MUSF) were clearly segregated from the two male groups, and a remarkable inter-group distance was also observed between MUBF and MUSF. This distribution pattern suggests that sex and body size exert a stronger effect on the volatile metabolite profiles of female crayfish muscle. The first two principal components (PC1 and PC2) of the PCA cumulatively accounted for 35.3% of the total variance. Although this proportion of explained variance was relatively modest, a pronounced separation trend across all experimental groups was clearly discernible in the multivariate projection (Figure 1B). To further corroborate the clustering patterns identified by PCA, sparse partial least squares discriminant analysis (sPLS-DA) was conducted as a complementary multivariate approach (Figure 1C). The sPLS-DA results yielded inter-group clustering patterns congruent with those observed in PCA, while samples within each treatment group exhibited tighter intra-group aggregation. The first and second components of the sPLS-DA model explained 13.1% and 20.9% of the total variance, respectively (Figure 1C). This variance profile reflects an inherent trade-off between the model’s total variance explanatory capacity and its class discrimination performance, and the multivariate analytical models employed in this study demonstrated acceptable reproducibility (Figure 1B, C).

Meanwhile, the hierarchical clustering dendrogram further corroborated the aforementioned PCA results. The dendrogram also indicated that the volatile profile of small-sized female crayfish muscle (MUSF) exhibited the greatest divergence among the four groups, forming an independent branch distinct from the other three clusters (Figure 1D). Furthermore, one-way analysis of variance (ANOVA) identified 56 volatile metabolites with significantly different relative abundances across the four experimental groups. The differential metabolites were visualized through a heatmap (Figure 1E), which intuitively showed variations in metabolite levels among different muscle sample groups.

**Figure 2:**
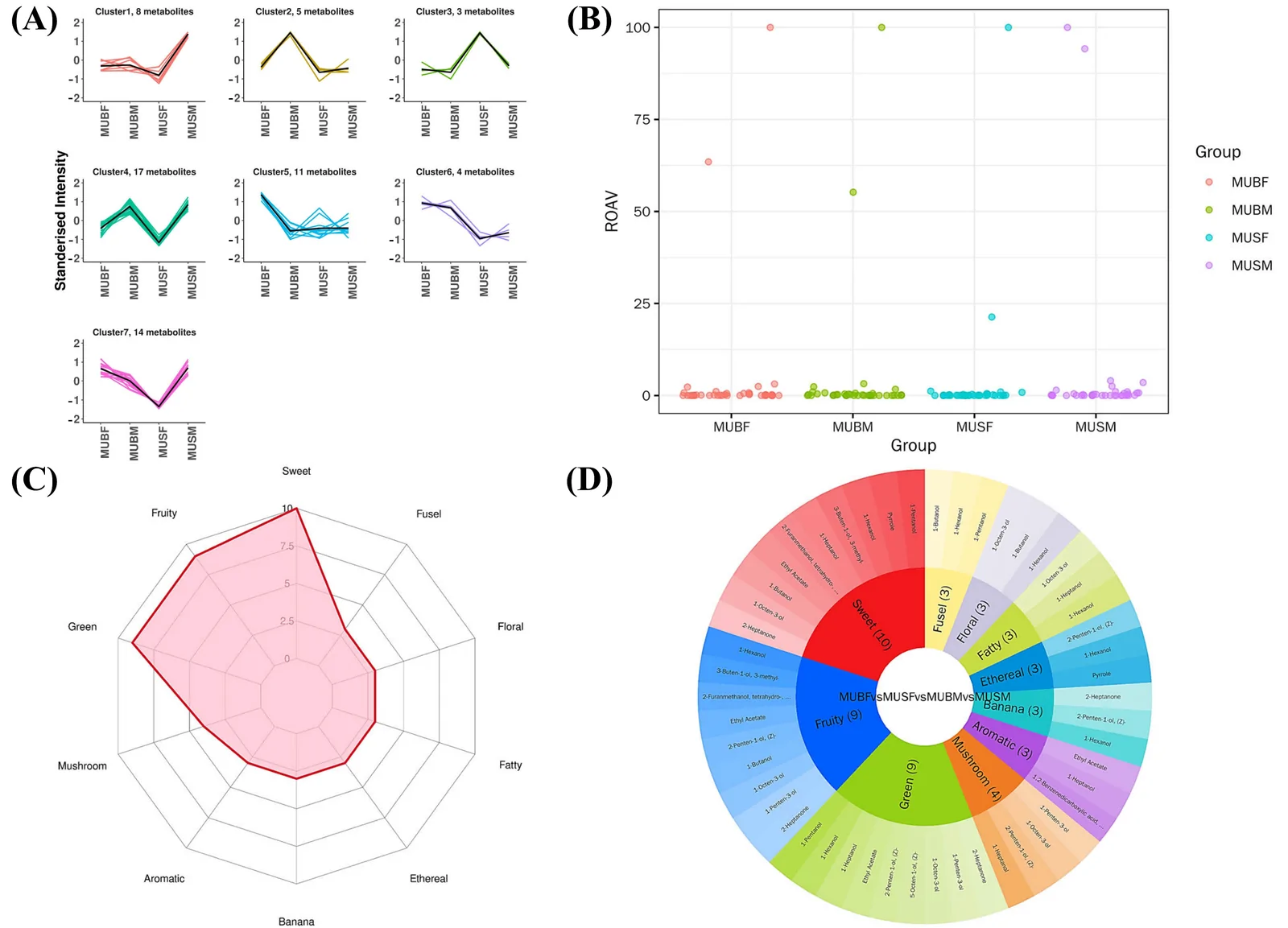
K-means clustering analysis (**A**), scatter plot analysis of ROAV (**B**), radar chart analysis of flavors (**C**), and flavor wheel chart analysis (**D**) for MUBF, MUBM, MUSF and MUSM groups.

Additionally, K-means clustering was performed to visualize dynamic inter-group variations in the detected and identified volatile metabolites. Based on their variation trends across experimental groups, the metabolites were categorized into seven distinct clusters (Figure 2A), containing 8, 5, 3, 17, 11, 4, and 14 compounds in Clusters 1–7, respectively. Figure 2A showed the fluctuation profiles of their relative abundances across the four experimental groups (MUBF, MUBM, MUSF, and MUSM), demonstrating that different combinations of body size and sex exert dynamic and complex influences on the accumulation of volatile metabolites in crayfish muscle.

### 3.4. Key Vocs via Roav Analysis

Volatile organic compounds (VOCs) in crayfish muscle samples were profiled via headspace solid-phase microextraction coupled with gas chromatography–mass spectrometry (HS-SPME-GC-MS). By cross-referencing mass spectral data against established odor threshold databases, a total of 141 volatile constituents were positively identified across all experimental groups, including 5 volatile compounds identified only by name without other information. Relative odor activity value (ROAV) analysis was applied to quantify the aroma contribution of each identified compound. In total, 38 aroma-active components were detected in each of the four treatment groups. Specifically, 19, 18, 12, and 20 compounds were categorized as important odor components (ROAV > 0.1, Table 2) in the MUBF, MUBM, MUSF, and MUSM groups, respectively, while the corresponding counts of key odor components were 6, 5, 3, and 8. Complete ROAV profiles are tabulated in Table 2 and Figure 2B. Ethanol was the ROAV-maximum (ROAV_max_ = 100) odor compound in the MUBF, MUBM, and MUSF groups, whereas 1-octen-3-ol was the dominant ROAV contributor in the MUSM group. Chemically, the confirmed important and key odor components fell into five distinct classes: 8 alcohols, 8 aldehydes, 3 ketones, 1 ester, and 1 aromatic hydrocarbon. Notably, 12 important or key odor volatile compounds (ROAV > 0.1) were present across all four sample groups: ethanol, 1-pentanol, 1-hexanol, 1-octen-3-ol, 1-heptanol, benzyl alcohol, 2-methylbutanal, hexanal, nonanal, benzaldehyde, ethyl acetate, and styrene (Table 2).

Overall, the volatile odor profiles of crayfish muscle across the four experimental groups were predominantly characterized by the sweet sensory attribute, followed by fruity and green flavor (Figure 2C). Specifically, ten odor compounds were classified under the sweet attribute, while nine compounds fell into the fruity and green flavor categories, respectively. Four compounds were attributed to the mushroom flavor attribute, and each remaining flavor class comprised three corresponding odor compounds (Figure 2D).

### 3.5. Different Compounds in Muscles Derived from Different Body-Size or Gender Groups

**Figure 3:**
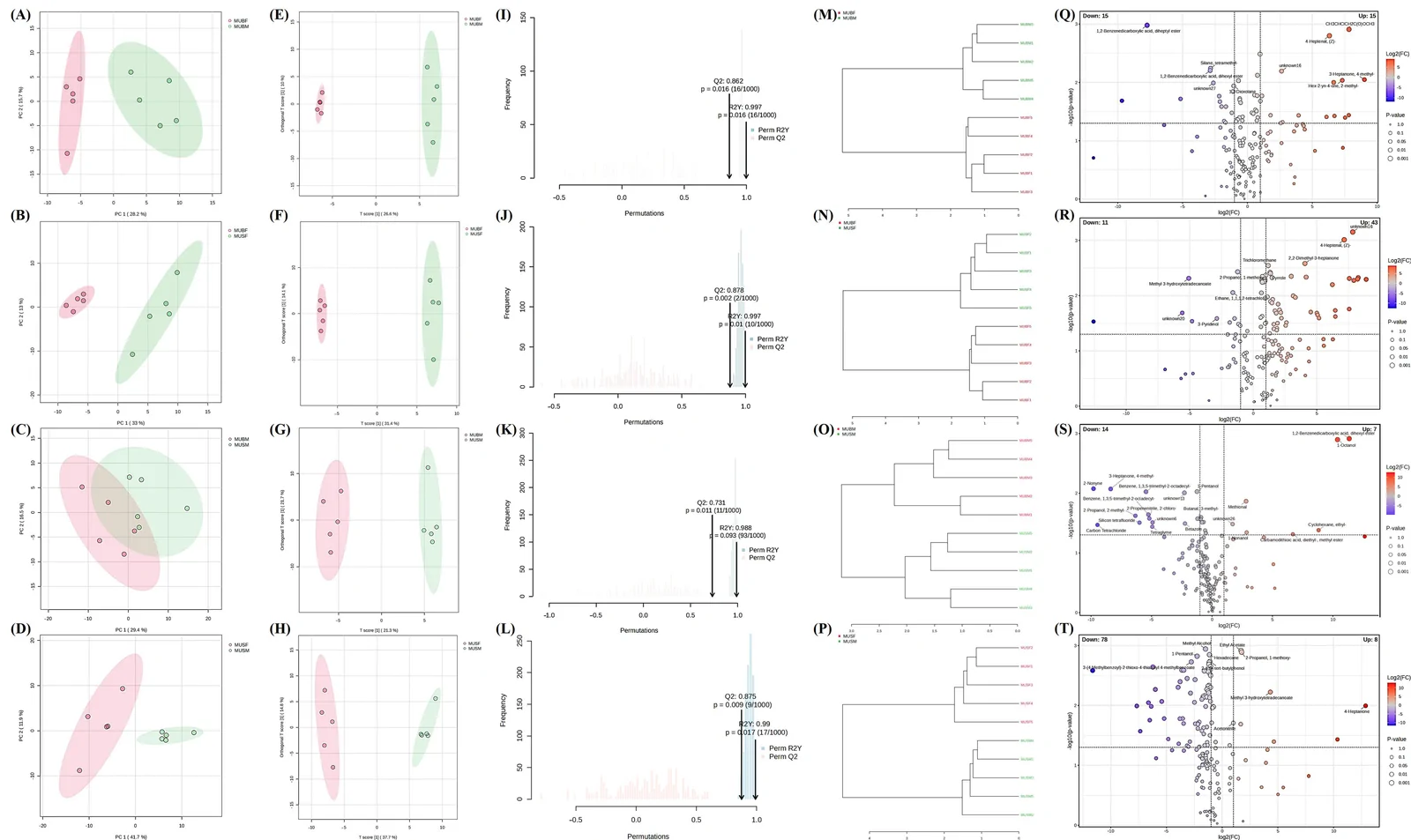
Multivariate statistical analysis of volatile compound profiles based on two sets of inter-group comparisons: sex-based differences within the same body size class (MUBF and MUBM, MUSF and MUSM), and size-based differences within the same gender (MUBF and MUSF, MUBM and MUSM) for *C. quadricarinatus* muscle samples. (**A**,**B**,**C**,**D**) PCA score plot showing the spatial distribution in the pairwise comparison of MUBF and MUBM, MUBF and MUSF, MUBM and MUSM, and MUSF and MUSM, respectively; (**E**,**F**,**G**,**H**) oPLS-DA score plot presenting the distribution in the pairwise comparison of MUBF and MUBM, MUBF and MUSF, MUBM and MUSM, and MUSF and MUSM, respectively; (**I**,**J**,**K**,**L**) Permutation test for the oPLS-DA score plots, respectively; (**M**,**N**,**O**,**P**) Dendrogram plot showing the clustering outcomes and the degree of inter-group differentiation across the pairwise comparison of the MUBF and MUBM, MUBF and MUSF, MUBM and MUSM, and MUSF and MUSM, respectively; (**Q**,**R**,**S**,**T**) Volcano plot volcano depicting significantly upregulated and downregulated volatile metabolites in the pairwise comparison of MUBF and MUBM, MUBF and MUSF, MUBM and MUSM, and MUSF and MUSM, respectively.

Furthermore, a panel of multivariate statistical approaches consistently demonstrated distinct separation across the MUBF, MUSF, MUBM, and MUSM groups when stratified by sex and body size, respectively. Principal component analysis (PCA) scores plots (Figure 3A,B,C,D), orthogonal partial least squares discriminant analysis (OPLS-DA) models (Figure 3E,F,G,H) validated by corresponding permutation tests (Figure 3I,J,K,L), and hierarchical clustering dendrograms (Figure 3M,N,O,P) all corroborated the distinct grouping patterns. This robust discriminability across independent analytical frameworks confirms that the volatile metabolite profiles of crayfish muscle are characteristically modulated by both sex and body size.

For pairwise comparisons between sexes within the identical size grades, 25 differentially abundant volatile metabolites (DAVMs) were identified between the MUBF and MUBM groups, of which 16 displayed significantly elevated relative abundance and 9 exhibiting significantly reduced relative abundance (Figure 3Q, Table S1). Parallel analysis of the MUSF and MUSM groups revealed 41 DAVMs, with 40 significantly upregulated and only 1 significantly downregulated in the MUSF group (Figure 3R, Table S2). This striking disparity in the count of DAVMs indicates that body size exerts a less pronounced modulatory effect on the volatile metabolite composition of muscle tissue in male crayfish relative to female conspecifics.

With respect to size-based pairwise comparisons within each sex, 30 DAVMs were uncovered between the MUBF and MUSF groups. Remarkably, all 30 metabolites exhibited significantly lower abundance in MUSF, whereas no metabolites were found to be significantly enriched in this group (Figure 3S, Table S3). In male crayfish, by contrast, only 3 DAVMs were detected between the MUBM and MUSM groups, including 1 up-regulated and 2 down-regulated metabolites (Figure 3T, Table S4). Collectively, these findings demonstrate that body size drives a greater degree of compositional divergence in muscle volatile metabolite profiles among female crayfish compared with male counterparts.

### 3.6. Metabolic Pathway Enrichment Analysis of the Volatile Metabolites in Muscle of C. quadricarinatus

**Figure 4:**
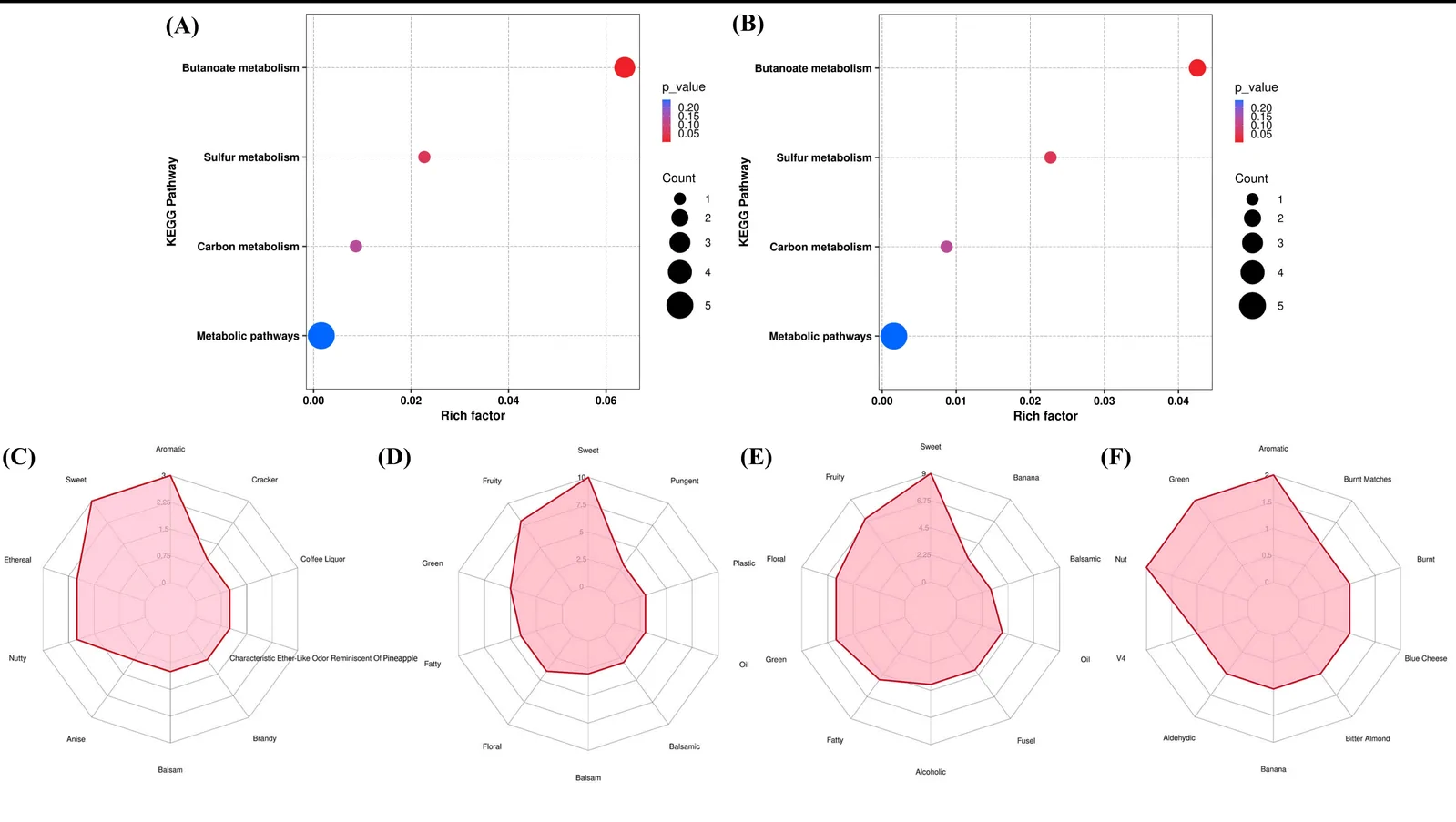
KEGG Pathway Enrichment Analysis of pairwise inter-group comparisons of MUBF and MUSF (**A**), and MUSF and MUSM (**B**), and the radar plot analysis of flavor characteristic profiling of differential metabolites of pairwise inter-group comparisons of MUBF and MUBM (**C**), MUBF and MUSF (**D**), MUBM and MUSM (**E**), and MUSF and MUSM (**F**), respectively.

Following pairwise inter-group comparisons across two analytical dimensions (sex-based comparisons at equivalent size grades and size-based comparisons within the same sex), Kyoto Encyclopedia of Genes and Genomes (KEGG) pathway enrichment analysis was conducted on the DVAMs. No statistically significant enrichment of metabolic pathways was detected in either the large female and male (MUBF and MUBM) comparison or the large male and small male (MUBM and MUSM) comparison. In contrast, the differentially abundant metabolites identified from the pairwise contrasts of MUSF and MUSM, and MUBF and MUSF were mapped to four significantly enriched metabolic pathways (Figure 4): Metabolic pathways (cqd01100), Butanoate metabolism (cqd00650), Carbon metabolism (cqd01200), and Sulfur metabolism (cqd00920). Notably, Butanoate metabolism (cqd00650) crossed the threshold of statistical significance in both the contrasts of MUSF and MUSM (*p* value = 0.00120, Figure 4A) and MUBF and MUSF (*p* value = 0.00001, Figure 4B). Especially, metabolites annotated to the butanoate metabolism pathway were significantly upregulated in the MUSF group relative to the MUSM group, but significantly downregulated in MUBF relative to MUSF. This concordant regulatory pattern suggests that butanoate metabolism is maintained at a relatively high level of metabolic activity in the muscle tissue of small female crayfish.

### 3.7. Differential Odor-Active Compounds Across Size and Sex Groups

Consistent with the metabolomic analysis framework, intergroup comparisons of sensory attributes were conducted across the same two dimensions: sex-based contrasts at matched size grades and size-based contrasts within each sex. Regarding the sensory flavor profiles inferred from DAVMs, moderate divergence was observed between large female (MUBF) and large male (MUBM) crayfish, primarily driven by differences in aromatic, sweet, ethereal, and nutty odor attributes (Figure 4C). By contrast, more pronounced divergences in potential sweet, fruity, green, fatty, and floral flavor attributes were detected between small female (MUSF) and small male (MUSM) crayfish (Figure 4D). Taken together, sex-based flavor variation was relatively limited in the large-sized cohort but markedly more prominent in small-size individuals. For size-based comparisons within each sex, clear differences in sweet, fruity, floral, green, and fatty odor notes were identified between the MUBF and MUSF groups (Figure 4E). By contrast, only subtle variations in potential aromatic, green, and nutty flavor attributes were observed between the MUBM and MUSM groups (Figure 4F).

Among the 21 key odor-active volatile compounds (ROAV ≥ 0.1) identified and quantified in the present study (Table 2), pairwise comparisons across size grades within each sex and between sexes at matched size grades revealed heterogeneous distribution patterns of these compounds in crayfish muscle across all experimental groups. Ten of these odor-active compounds showed no statistically significant differences across all aforementioned group comparisons, suggesting that they may serve as baseline volatile flavor constituents in crayfish muscle, including ethanol, 1-penten-3-ol, (Z)-5-octen-1-ol, 2-methylbutanal, hexanal, heptanal, (Z)-4-heptenal, octanal, benzaldehyde, and 2-octanone. On the other hand, the remaining 11 volatile flavor metabolites exhibited significant disparities in abundance in at least one pairwise comparison, implying their potential roles as discriminative flavor markers underlying flavor variations between sexes at the same size or between size groups within the same sex.

**Figure 5:**
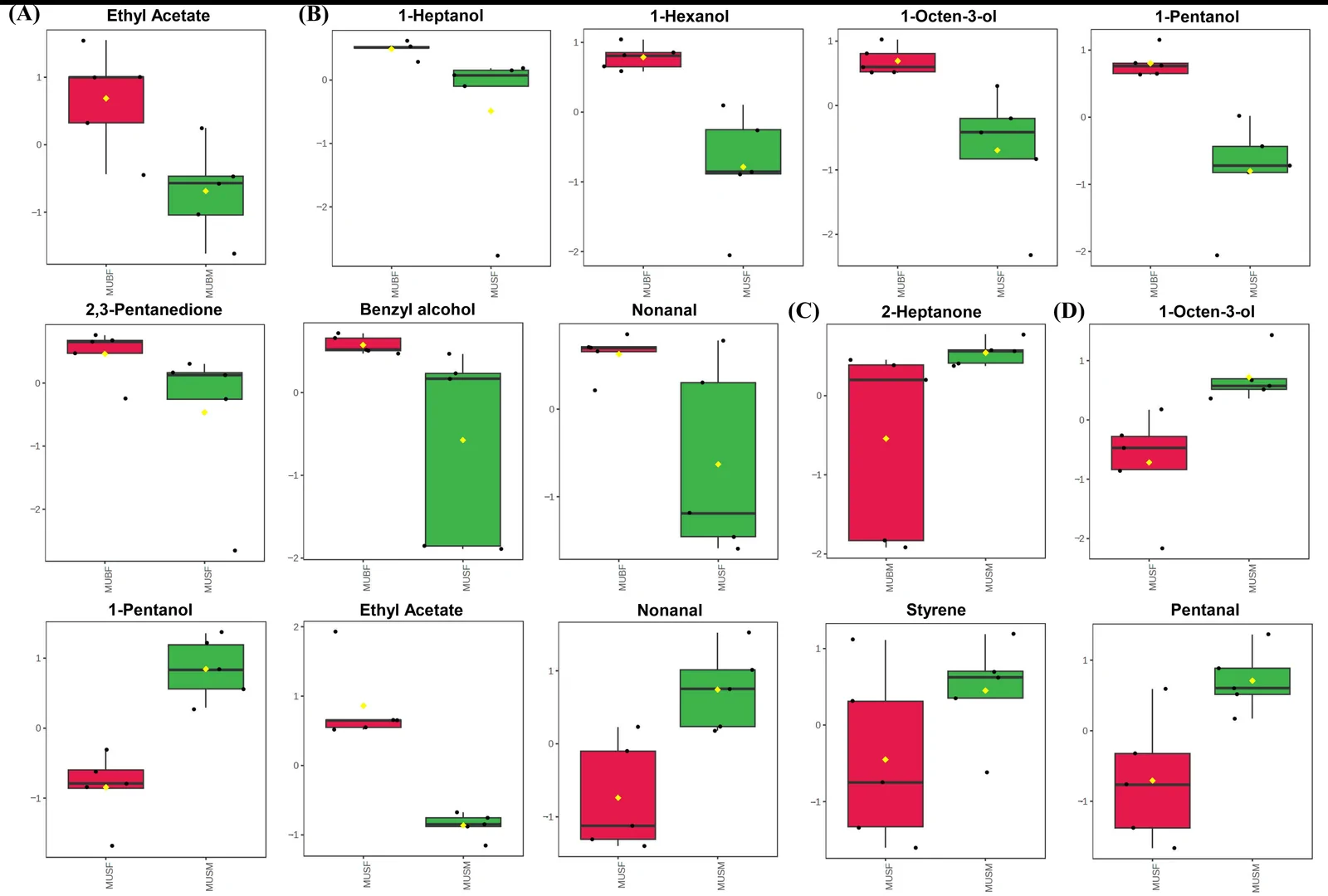
The significantly different metabolites (*p* value < 0.05 and VIP > 1) from pairwise comparisons exerting critical regulatory effects on overall flavor perception (ROAV > 0.1). (**A**) Metabolites from the pairwise comparison between MUBF and MUBM; (**B**) Metabolites from the pairwise comparison between MUBF and MUSF; (**C**) Metabolites from the pairwise comparison between MUBM and MUSM; (**D**) Metabolites from the pairwise comparison between MUSF and MUSM. The yellow diamond markers represent the arithmetic mean of each group, and the Black dots represent individual data points of biological replicates. The solid black line within the box indicates the median (50th percentile). The upper and lower edges of the box correspond to the 75th percentile (upper quartile, Q3) and 25th percentile (lower quartile, Q1), respectively.

Specifically, for large-sized crayfish, one odor-active compound was significantly downregulated in the MUBF group relative to the MUBM group (Figure 5A), indicating that it may act as a key contributor driving the volatile flavor divergence between large male and large female crayfish. Correspondingly, large male crayfish exhibited more intense odor profiles characterized by sweet, aromatic, anise, balsam, and brandy notes, compared with their female counterparts. For small-sized crayfish, six differentially abundant odor-active compounds were detected between the MUSF and MUSM groups, of which five were significantly upregulated and one was significantly downregulated in female individuals (Figure 5B). The upregulated compounds were predominantly associated with fatty, sweet, grassy, oily, soapy, fruity, rose/floral, balsam, and earthy odor characteristics, while the downregulated compound mainly contributed to sweet, aromatic, anise, and brandy-like flavors. This finding demonstrates that marked volatile flavor differences exist in muscle between small male and small female crayfish, and the flavor profile of small-sized females is more diverse and compositionally complex.

With respect to size-based comparisons within each sex, a total of seven differentially abundant flavor compounds were identified between the MUBF and MUSF groups, all of which were significantly downregulated in MUBF relative to MUSF (Figure 5C). These differential compounds were primarily associated with a broad spectrum of odor attributes encompassing sweet, fatty, fruity, grassy, nutty, rose/flower, pungent, green, soapy, and earthy notes. This result indicates that large female crayfish possess a simpler flavor composition relative to small females, whereas the muscle of small female crayfish is distinguished by a wider repertoire of flavor compounds at higher concentrations. For male crayfish, only one differentially abundant flavor compound was observed between the MUBM and MUSM groups, with its concentration significantly elevated in the MUBM group (Figure 5D). This compound primarily imparts a fruity odor note, suggesting that large male crayfish may have a more homogeneous yet distinctive flavor profile compared with small-sized conspecifics.

### 3.8. Correlation Analysis of Volatile Components in Muscle

**Figure 6:**
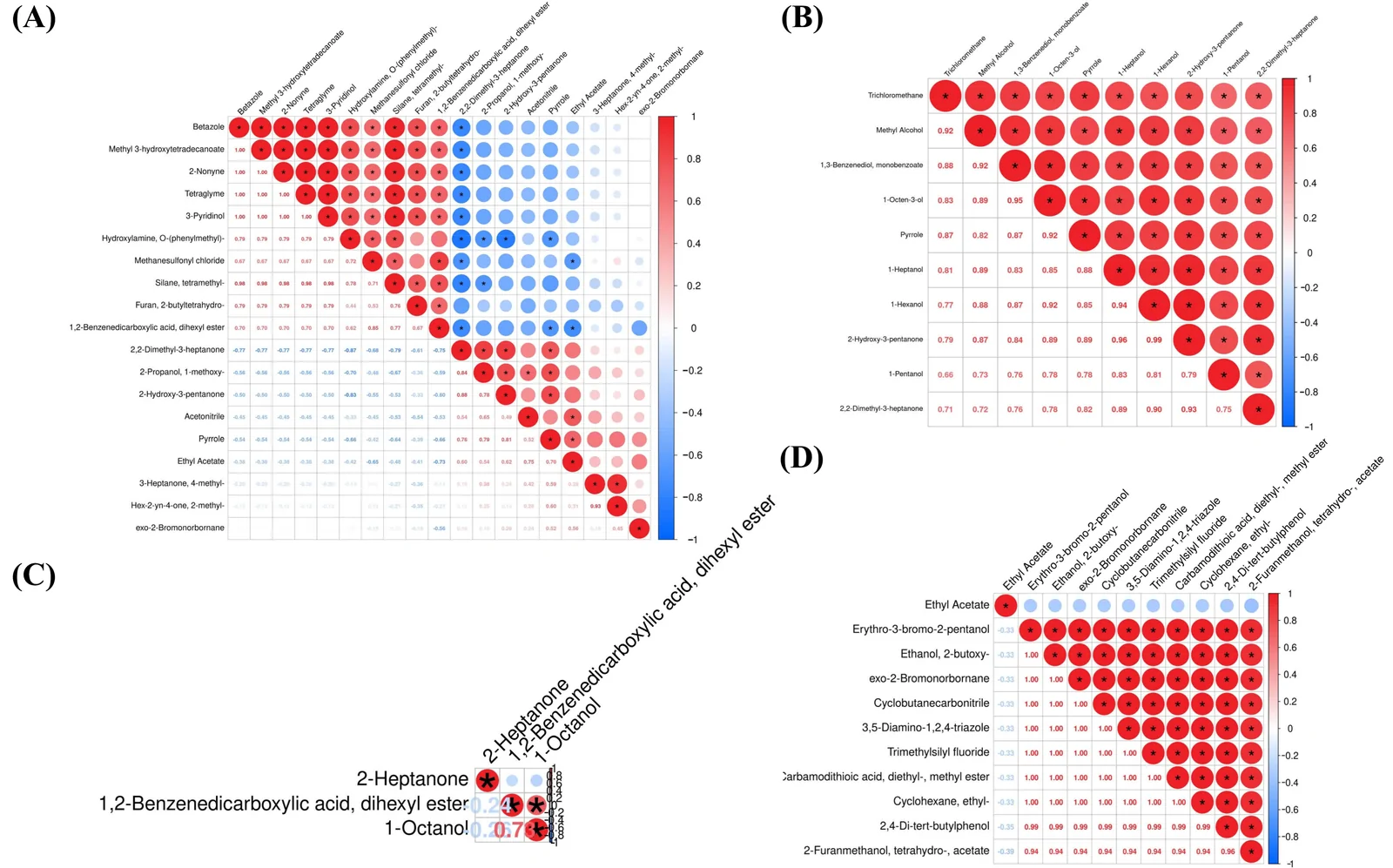
Heatmap of correlation analysis for the pairwise comparison of MUBF and MUBM (**A**), MUBF and MUSF (**B**), MUBM and MUSM (**C**), and MUSF and MUSM (**D**). *, *p* value < 0.05.

Pearson correlation analysis was conducted to elucidate the interrelationships among volatile flavor compounds in crayfish muscle samples. The correlation matrix was visualized as a heatmap in Figure 6, where red gradients represent positive correlations and blue gradients represent negative correlations. The analysis revealed a consistent covariation pattern across all pairwise comparison groups: a distinct cluster of volatile metabolites showed consistent correlation trends relative to the remaining compounds, which was consistent with the between-group differences described in previous sections. For the MUBF and MUBM comparison (Figure 6A), a panel of compounds including betazole, methyl 3-hydroxytetradecanoate, 2-nonyne, tetraglyme, 3-pyridinol, O-benzylhydroxylamine, methanesulfonyl chloride, tetramethylsilane, 2-butyltetrahydrofuran, and 1,2-benzenedicarboxylic acid dihexyl ester formed a tightly cohesive cluster characterized by pervasive significant positive intra-cluster correlations. Conversely, this cluster exhibited widespread negative correlations with another set of volatiles: 2,2-dimethyl-3-heptanone, 1-methoxy-2-propanol, 2-hydroxy-3-pentanone, acetonitrile, pyrrole, ethyl acetate, 4-methyl-3-heptanone, 2-methylhex-2-yn-4-one, and exo-2-bromonorbornane. Analogous correlation profiles were consistently observed in the MUBF and MUSF (Figure 6B), MUBM and MUSM (Figure 6C), and MUSF and MUSM (Figure 6D) comparisons. The high conservation of this covariation pattern across independent contrasts strongly suggests that the aforementioned metabolite cluster may share common biosynthetic origins or be tightly coupled via interconnected metabolic pathways.

Beyond conserved cluster-level covariation, both universal and size-specific correlation patterns were discerned among individual volatile flavor compounds. As a representative conserved cross-group association, pyrrole displayed a significant positive correlation with 2,2-dimethyl-3-heptanone in both the MUBF and MUBM and MUBF and MUSF comparisons (Figure 6A,B). In contrast, size-dependent reversals in correlation direction were also detected. For instance, ethyl acetate and exo-2-bromonorbornane exhibited a positive correlation in the MUBF and MUBM pair but a negative correlation in the MUSF and MUSM pair. This directional reversal indicates that the interrelationships between volatile flavor compounds in crayfish muscle may shift from a synergistic to an antagonistic state as body size increases (Figure 6A,D).

### 3.9. Multi-Level Metabolic Interaction Network Analysis

**Figure 7:**
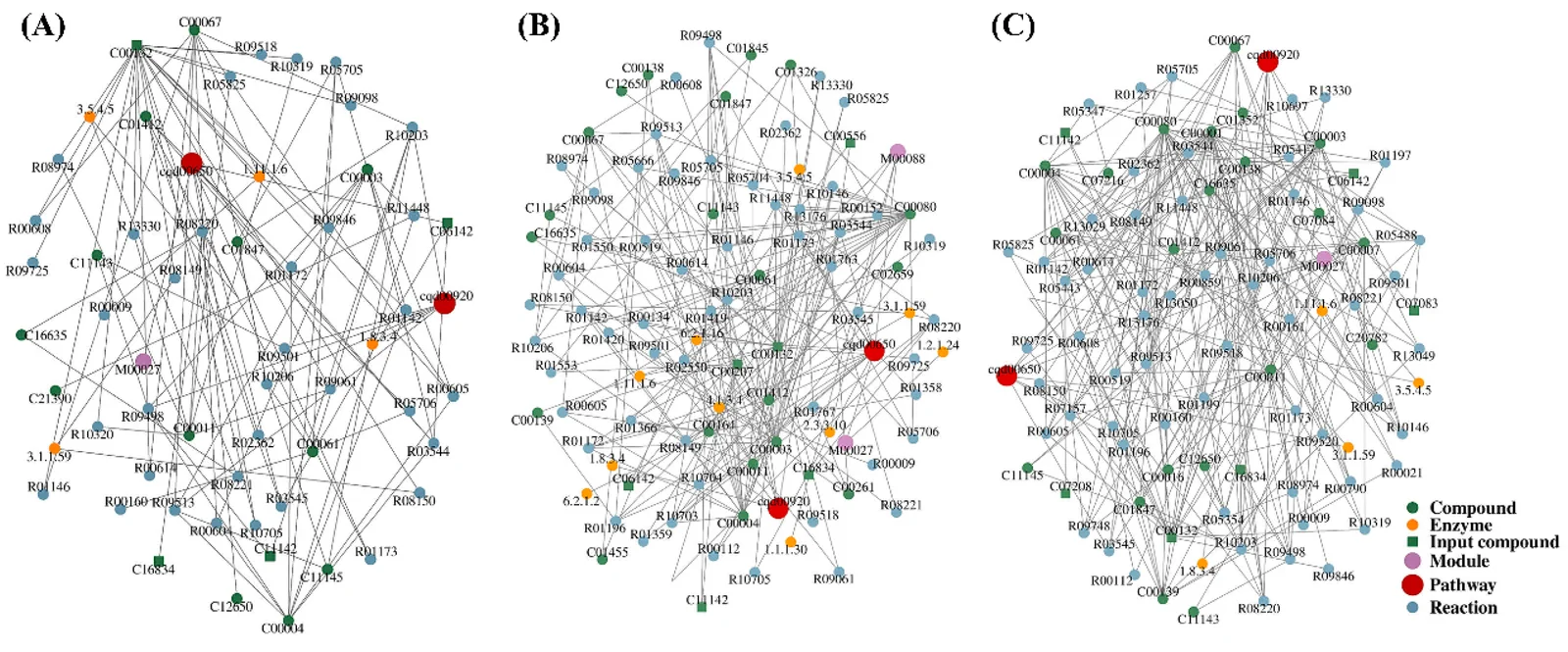
Network analysis for the multi-group comparison of MUBF, MUBM, MUSF and MUSM (**A**), the pairwise comparison of MUBF and MUSF (**B**), and the pairwise comparison of MUSF and MUSM (**C**).

To further dissect the interrelationships and intrinsic molecular crosstalk underlying volatile flavor metabolite profiles, multilevel interaction networks integrating metabolic pathways, biochemical reactions, functional modules, enzymes, and metabolites were constructed at two analytical tiers. Specifically, a global network was generated using pooled data from all four experimental groups (MUBF, MUBM, MUSF, and MUSM; Figure 7A), and separate pairwise networks were built for the previously described two-group comparisons (MUBF and MUSF, Figure 7B; MUSF and MUSM, Figure 7C). Notably, robust interaction networks could not be established for the MUBF and MUBM, and MUBM and MUSM pairwise comparisons.

The global network derived from the full four-group dataset revealed that butanoate metabolism (cqd00650) and sulfur metabolism (cqd00920) formed a cross-linked functional architecture bridged by shared intermediate metabolites and coupled biochemical reactions. The γ-aminobutyrate (GABA) shunt (M00027) was identified as a core functional module embedded in this network. Methanol (C00132) and formaldehyde (C00067) served as the most critical hub metabolites, orchestrating metabolic flux across multiple branched metabolic pathways. As a canonical redox coenzyme pair, nicotinamide adenine dinucleotide (NAD^+^, C00003) and its reduced form (NADH, C00004) were inferred to exert pivotal regulatory control over redox homeostasis across all interconnected metabolic routes. Additionally, four rate-limiting enzyme nodes, including catalase (EC 1.11.1.6), cytidine deaminase (EC 3.5.4.5), juvenile hormone esterase (EC 3.1.1.59), and methanethiol oxidase (EC 1.8.3.4), modulated metabolic reactions in discrete pathway branches and collectively mediated the conversion of primary substrates to terminal metabolites within the network.

In the pairwise network comparing female crayfish across two size classes (MUBF and MUSF), the backbone metabolic pathways, key functional modules, and redox coenzyme pair were largely congruent with those observed in the global network. Nevertheless, methanol (C00132), carbon dioxide (C00011), and proton (C00080) emerged as the predominant core hub metabolites in this comparison, and ketone body biosynthesis (M00088) was identified as an additional crucial metabolic module. For the sex-based pairwise comparison between mature female and male crayfish (MUSF and MUSM), the network also exhibited conserved core pathways, regulatory modules, and redox coenzyme systems relative to the global four-group network. By contrast, the central hub metabolites in this network comprised methanol (C00132), oxidized ferredoxin (C00139), carbon dioxide (C00011), proton (C00080), and water (C00001).

Taken together, these findings demonstrate that pairwise multilevel interaction networks allow resolution of more granular, group-specific metabolic signatures and core hub metabolites, whereas the fundamental metabolic architecture, regulatory modules, biochemical reaction cascades, and cofactor systems remain conserved across all analytical scales. These multilevel interaction networks provide an intuitively visualize the coordinated metabolic features of target pathways and offer visual evidence for elucidating the biosynthetic mechanism underlying flavor precursors in crayfish muscle.

## 4. Discussion

Global capture fisheries and aquaculture production reached a record 235 million tonnes in 2024, of which 195 million tonnes were aquatic animal products. Approximately 89% of this aquatic animal output was allocated for direct human consumption, underscoring the significant role of fisheries and aquaculture in supporting global food security [20].

Ongoing advances in detection and analytical technologies have enabled comprehensive qualitative and quantitative profiling of odor volatile compounds (OVCs) across a growing spectrum of aquatic food products. The functional roles of OVCs in flavor biogenesis and throughout the production and processing chain have been progressively elucidated. This body of literature has become a key foundation foroptimizing processing technologies and parameters of aquatic products, as well as for the development of targeted flavor regulation [50,51]. Beyond flavor- centric applications, OVC profiling serves as a sensitive, non-destructive analytical tool for freshness monitoring and authenticity traceability of raw seafood, both of which represent core quality control demands across aquatic product supply chains [52,53]. In this context, systematic qualitative and quantitative characterization of muscle OVCs in species suitable for raw consumption potential, such as *C. quadricarinatus*, provides essential baseline data to underpin product quality grading, selective breeding of elite germplasms, and the development of high-value ready-to-eat raw product lines.

In the present study, pooling muscle tissue from 5 individual crayfish per replicate possibly diminishes the capacity to evaluate individual biological variation. Nevertheless, pooling multiple individuals to form replicate samples constitutes a deliberate methodological trade-off. On one hand, it reduces inter-individual biological variability and enhances the statistical power to detect group-level effects. On the other hand, it may mask individual differences and result in the loss of information from outlier samples. In the present study, our core objective was to characterize the differences in muscle flavor profiles of red-claw crayfish associated with different size grades and sexes. Given that the study focused on population-level group effects rather than individual variation, pooled sampling was deemed appropriate, as it attenuates the impact of random individual differences on the overall population characteristics. Similar sampling approaches have been adopted in prior studies, supporting the feasibility of this method to a certain extent [54,55].

Moreover, the present study primarily investigates the volatile compounds from a chemical perspective. Beyond classifying and profiling the experimental groups of different sizes and sexes based on the composition and features of the overall volatile metabolome, this study generally focuses on describing and analyzing the compositional characteristics of representative volatile flavor-associated metabolites and their corresponding metabolic pathways across groups stratified by size grade and sex.

Juvenile crayfish with uniform initial sizes were reared under standardized aquaculture conditions for an identical rearing period. With body size and sex designated as experimental variables, four treatment groups were established: large female (BF), large male (BM), small female (SF), and small male (SM) crayfish. Volatile metabolites in the muscle tissue of each group were detected and profiled to delineate the compositional basis and chemical diversity of volatile odor compounds in crayfish muscle.

Overall, significant size-dependent variations were observed in crude protein, crude lipid, moisture content, and meat yield across all experimental groups. No significant sex-associated differences were detected in any parameters. These findings are consistent with previous reports. As individual body size increases, meat yield, crude protein content, crude fat content and other proximate composition indicators generally exhibit a pronounced downward trend [56].

Using HS-SPME/GC-MS, a total of 172 volatile metabolites were detected in the muscle samples, 136 of which were successfully annotated. The identified compounds were classified into 14 chemical categories: alcohols, heterocyclic compounds, acids, ketones, aldehydes, esters, halogenated hydrocarbons, ethers, amines, organic nitrogen-containing compounds, phenols, aromatic hydrocarbons, organic sulfur-containing compounds, and miscellaneous volatile metabolites. This compositional profile indicates relatively high diversity and structural complexity of volatile compounds in crayfish muscle. Multivariate statistical analysis further revealed distinct OVC profiles across treatment groups, and these variations appeared to be modulated by both body size and sex.

The flavor of aquatic food products is largely governed by the combined and interactive effects of volatile and non-volatile compounds. Volatile flavor compounds, predominantly aldehydes, esters, and alcohols, contribute a diverse spectrum of aroma attributes including seafood-like, sweet, green, grassy, and fruity notes. Accordingly, volatile compounds are widely acknowledged as a critical determinant of the overall flavor profile of aquatic products [57].

In this study, alcohols represented the most abundant class of volatile metabolites, with 28 distinct compounds detected in total. Eight alcohol metabolites showing significant concentration differences were further identified as key odor-active compounds based on a predefined screening threshold (ROAV value ≥ 0.1). As one of the major groups of OVCs, alcohols generally impart pleasant aromas to aquatic products, with dominant flavor attributes including sweet, grassy, fatty, and earthy notes. Saturated linear and branched alcohols, exemplified by ethanol and 1-pentanol, are typically generated via lipid oxidation pathways [58,59]. Saturated alcohols such as 1-hexanol and 1-heptanol are generally r considered minor contributors to overall odor perception due to their relatively high odor thresholds. When their concentrations exceed respective threshold values, however, these compounds may impart grassy and green flavor characteristics to aquatic food products [60]. Aromatic alcohols, represented by benzyl alcohol, are widely reported to be formed via amino acid degradation. Due to their high odor thresholds, these compounds do not typically act as key OVCs in aquatic products. Nevertheless, at concentrations above the sensory threshold, they may add rose-like floral nuances to the overall flavor profile [50].

Unsaturated alcohols identified in this study, including 1-penten-3-ol, 1-octen-3-ol, and (Z)-5-octen-1-ol, exhibit comparatively lower sensory thresholds and accordingly make more substantial contributions to the flavor of aquatic products. These compounds mainly present plant-like aromas commonly described as grassy, sweet, mushroom, and earthy-like notes [50]. Notably, 1-penten-3-ol and 1-octen-3-ol are the most prevalent branched-chain alcohols reported in aquatic products. Excessive accumulation of these compounds may lead to the development of earthy off-flavors, making them a notable component of the volatile aroma profile that warrants targeted attention [61]. In the current work, 1-octen-3-ol was identified as one of the flavor markers distinguishing female *C. quadricarinatus* across size groups (large female crayfish, MUBF; small female crayfish, MUSF). The concentration of 1-octen-3-ol in muscle tissue was higher in larger female crayfish than in small individuals. This positive association with favorable flavor attributes suggests the potential of large female *C. quadricarinatus* as a candidate for raw-ready aquatic food products. However, caution should be exercised in subsequent aquaculture practices or selective breeding programs to prevent excessive accumulation of this compound that could elicit undesirable earthy off-flavors.

Accumulating evidence demonstrates that aldehydes constitute the most abundant class of OVCs in aquatic food products and serve as major contributors to overall flavor profiles. Even at relatively low concentrations, these compounds can exert pronounced effects on the characteristic flavor of food matrices. As signature OVCs in aquatic matrices, aldehydes are predominantly derived from oxidative degradation of lipids [62]. Straight-chain saturated and unsaturated aldehydes with 3–4 carbon atoms are associated with strong pungent odors. Those with 5–9 carbon atoms tend to impact clean, fatty, and oily notes, whereas aldehydes with 10–12 carbon atoms typically exhibit lemon and orange peel-like aromas [33].

In the present study, eight aldehyde metabolites were identified as important contributors to the overall muscle aroma of crayfish, with ROAV values above 0.1. Among these, 2-methylbutanal is a 5-carbon branched saturated aliphatic aldehyde associated with balsamic notes. Pentanal, hexanal, heptanal, octanal, and nonanal are straight-chain saturated aliphatic aldehydes with 5 to 9 carbon atoms. (Z)-4-heptenal, an unsaturated aliphatic aldehyde, predominantly exhibits fatty, fishy, and flavor attributes. These aliphatic aldehydes are primarily derived from auto-oxidation of unsaturated fatty acids such as oleic acid and linoleic acid in aquatic products [50], a pathway consistent with the relatively high content of unsaturated fatty acids reported in crustacean muscle. Benzaldehyde, by contrast, is an aromatic aldehyde with nutty and metallic notes, which is presumably generated via amino acid degradation [63].

Aldehydes are major sources of grassy, nutty, and fatty flavors in aquatic products, whereas excessive concentrations may induce fishy off-flavors. No significant differences in total aldehyde content were observed between large-sized female and male crayfish (MUBF and MUBM), nor between large-sized and small-sized male crayfish (MUBM and MUSM). Such patterns suggest comparable volatile flavor profiles between male and female crayfish at larger size grades. Nevertheless, the content of nonanal differed significantly between large-sized and small-sized female crayfish (MUBF and MUSF), as well as between small-sized female and male crayfish (MUSF and MUSM). The concentration of nonanal in the muscle of small-sized female crayfish was notably higher than in the other two groups. Similarly, pentanal content differed significantly between small-sized female and male crayfish (MUSF and MUSM), with higher values detected in small-sized females relative to large-sized females. These observations indicate that aldehydes tend to accumulate more readily in the muscle tissue of smaller female individuals. Their concentrations may decline gradually with crayfish growth, and sex-based differences may diminish as body size increases. Furthermore, longer-chain aldehydes may exhibit greater retention as characteristic flavor compounds.

Ketones are generated via multiple metabolic pathways, including oxidative degradation of polyunsaturated fatty acids, amino acid catabolism, microbial metabolism, and β-oxidation of lipids; these compounds generally impart fatty and roasted notes to food matrices [64,65]. Three ketone compounds with ROAV values ≥ 0.1 were detected in the present study, namely 2,3-pentanedione, 2-heptanone, and 2-octanone. All three are straight-chain saturated aliphatic ketones (2,3-pentanedione is a diketone), and predominantly exhibit sweet and fruity-like flavor characteristics. Relatively few ketone species have been reported in aquatic products. For instance, 2,3-octanedione is associated with fatty, milky, green, and grassy flavors, and contributes grassy and green notes to large yellow croaker and swimming crab (*Portunus trituberculatus*) [66,67]. 2, 3-pentanedione can serve as a lipid oxidation indicator in frozen fish muscle, enabling monitoring of quality deterioration caused by lipid oxidation [68]. In the current work, 2, 3-pentanedione content was significantly higher in the muscle of small-sized female crayfish compared with large-sized females, which suggests more active lipid oxidation reactions in the muscle of smaller individuals.

Esters are typically formed via esterification of short-chain free fatty acids with alcohols, or transesterification of triglycerides with ethanol. These compounds contribute fruity and floral aromas to aquatic products [69]. In this study, ethyl acetate content in the muscle of large-sized male crayfish was significantly higher than that in female crayfish of the same size grade, and also higher than that in small-sized males. Meanwhile, ethyl acetate concentrations in small male crayfish were also higher than those in females of corresponding size. These findings indicate size- and sex-dependent distribution of ethyl acetate in crayfish muscle, with higher levels in males than in females, and in larger individuals than small ones. Ethyl acetate has been reported to impart sweet notes to silver carp [61], and wine and pineapple-like flavors to large yellow croaker [66]. Its accumulation in the muscle of large-sized male crayfish may contribute to a more distinct flavor profile of the tissue.

Besides, only one aromatic hydrocarbon, styrene, was found to contribute to muscle flavor (ROAV value ≥ 0.1) in the present study. This compound mainly presents balsamic notes, and its concentration in the muscle of small female crayfish was significantly higher than that in small males (MUSF and MUSM). Aromatic hydrocarbons generally exhibit limited flavor activity and are not commonly considered key flavor compounds in aquatic products. However, styrene has been reported as one of the key OVCs in crucian carp (*Carassius auratus*) [70], and may play a role in shaping the aroma profile of small *C. quadricarinatus* muscle.

Additional classes of volatile compounds detected in the present study included organoheterocyclic compounds, acids, hydrocarbons, ethers, amines, halohydrocarbons, organic nitrogen compounds, phenols, organosulfur compounds, and others. The number of flavor-active compounds in each class was 5, 0, 3, 3, 1, 2, 1, 3, 1, and 1, respectively. None of these compounds met the ROAV screening threshold, indicating relatively limited contributions of these volatile metabolites to the overall volatile aroma of *C. quadricarinatus* muscle.

The flavor of aquatic animals is shaped by a multitude of factors, which account for the rich and highly variable flavor profiles across species and production systems. These factors include intrinsic biological properties of the animals, such as species, genetic background, growth stage, anatomical location, and nutritional status, as well as extrinsic factors including rearing environment, aquaculture mode, water quality, and feed sources.

The same aquatic species may exhibit flavor differences associated with cultivar traits, anatomical tissues, and growth stages. Accordingly, flavor profiling research can serve as an evaluation indicator for selective breeding, farming cycle optimization, edible tissue selection, and byproduct valorization. Such information can guide the breeding of elite varieties, upgrading of aquaculture modes, development of processing technologies, advancement of flavor development and byproduct refining systems.

Regarding aquaculture modes, consumers generally perceive wild fish to have better flavor than farmed fish [71]. Comparative studies on OVC profiles in the muscle of wild and farmed aquatic species have revealed distinct differences in types and concentrations of OVCs, with wild fish generally exhibiting superior flavor quality [50,72,73,74]. Therefore, the adoption of diversified aquaculture modes, such as recirculating aquaculture systems, raceway culture systems, hydroponic systems, paddy field farming systems, and photovoltaic-fishery integrated systems, may exert positive effects on the flavor quality of farmed aquatic animals [75].

Water quality represents a critical factor across all stages of aquaculture production. The OVCs in aquatic animals are modulated by key abiotic and biotic factors in rearing environment, including dissolved oxygen, total nitrogen, total phosphorus, chemical oxygen demand, and microbial communities. In *Micropterus salmonides*, tissue OVC concentrations are positively correlated with dissolved oxygen levels and negatively correlated with total nitrogen, total phosphorus, and chemical oxygen demand [50]. Grass carp reared in water with high nitrate and nitrite concentrations are prone to developing intense fishy off-flavors and reduced overall flavor quality [76].

Salinity is another key environmental factor modulating OVCs in mariculture species. Atlantic salmon reared in seawater versus freshwater exhibit significantly distinct OVC profiles across different growth stages [77]. Razor clams cultivated at high salinity show higher OVC abundances and more desirable flavor compared with those reared at low salinity [78]. Temporary depuration of oysters under varying salinity conditions also alters the composition and concentrations of OVCs, including alcohols, aldehydes, and ketones. A salinity of 29 g/L has been found to preserve desirable flavor attributes in *Crassostrea gigas*, suggesting that targeted regulation of aquaculture water salinity can enhance flavor quality [79]. Conversely, during salting processes of aquatic products such as crayfish, elevated salt levels can improve tissue firmness while inducing losses of characteristic flavor compounds [80].

Feed formulation represents another pivotal strategy for regulating OVC profiles in farmed aquatic animals. Manipulation of dietary composition during the grow-out phase is widely adopted to reduce off-flavor compounds and improve overall flavor quality [78]. For instance, high concentrations of specific flavor-active compounds in chilled feed may lead to corresponding accumulation in muscle of consuming animals. Varying protein sources in formulated feeds may modify composition and concentrations of OVCs in aquatic animals, and consequently shape overall flavor quality [50]. Partial replacement of fish meal with poultry by-product meal and krill meal as dietary protein sources has been proven to improve the flavor quality of *Litopenaeus vannamei* [79]. Studies on swimming crabs have also demonstrated that substitution of vegetable oil with fish oil in feed can elevate OVC concentrations and generate more desirable flavor profiles [80].

Metabolic pathway enrichment analysis yielded no significantly enriched functional terms in both the MUBF vs. MUBM and MUBM vs. MUSM comparisons. By contrast, four pathways were consistently and significantly enriched in the MUBF vs. MUSF and MUSF vs. MUSM comparisons, including Metabolic pathways, Butanoate metabolism, Carbon metabolism, and Sulfur metabolism. Functionally, Butanoate metabolism and Carbon metabolism are predominantly implicated in carbohydrate and lipid metabolic cascades, while Sulfur metabolism is tightly coupled with amino acid turnover. Previous research has well documented remarkable sexual dimorphism in crayfish metabolomic profiles. The metabolites driving these sex differences are mostly associated with basal metabolic rate, energy reserve mobilization, and stress responsiveness, and generally show higher endogenous levels in females [81]. In crustaceans, ovarian development strongly upregulates fatty acid biosynthesis, β-oxidation, and amino acid metabolic pathways in the hepatopancreas and muscle tissues. This endocrine-driven metabolic reprogramming is a core modulator of tissue metabolite profiles and may further shape flavor characteristics [82]. In addition, a transcriptomic study on Procambarus clarkii revealed that differentially expressed genes between fast- and slow-growing individuals are primarily enriched in metabolic pathways, such as arachidonic acid metabolism, glutathione metabolism, and alanine-aspartate-glutamate metabolism [83]. Such growth-related metabolic divergence may underlie the distinct pathway enrichment patterns observed across the size and sex groups in the present study.

Notably, Butanoate metabolism showed a coordinated expression pattern: it was significantly upregulated in the MUSF group but markedly downregulated in the MUBF group. This trend indicates that butanoate metabolic activity remains relatively high in small female red-claw crayfish and is closely associated with endogenous butanoate production, absorption, and metabolic flux. As a critical energy substrate, butanoate can be catabolized to carbon dioxide or acetyl-CoA via fatty acid oxidation pathways, and the generated acetyl-CoA further serves as a building block for de novo lipid and glucose synthesis. Specifically, butanoate-derived acetyl-CoA enters the tricarboxylic acid (TCA) cycle, is sequentially converted to citrate and oxaloacetate, and ultimately channels into the triose phosphate pathway to support gluconeogenesis [81]. Fatty acids, cholesterol, and ketone bodies are also recognized as downstream metabolites of the butanoate metabolic network. Recent evidence shows that inhibiting butanoate uptake and metabolism abrogates butanoate’s thermogenic effect in adipocytes, supporting its regulatory role in systemic energy homeostasis.

Taken together, butanoate likely acts as a central intermediate metabolite linking lipid metabolism and glycolysis/gluconeogenesis. The observed upregulation of butanoate metabolism in small females suggests enhanced lipid catabolism and glucose turnover. Conversely, in large female crayfish, lipids may be preferentially partitioned toward ovarian accumulation for reproductive investment, rather than being oxidized to meet immediate energy demands.

## 5. Conclusions

In summary, with strict control of seed stock, farming environment, nutritional conditions and culture period to minimize husbandry-related confounding effects, this study systematically characterized the volatile flavor profiles in the muscle of *Cherax quadricarinatus* across four groups stratified by body size and sex. A total of 136 structurally identified volatile metabolites (15 chemical categories) were detected, with alcohols, aldehydes, ketones, esters and aromatic hydrocarbons identified as the five dominant chemical classes contributing to muscle aroma. Overall, the crayfish muscle aroma was dominated by sweet-like notes, followed by fruity and green-like attributes.

The key phenotypic findings can be summarized as follows: sex-related differences in flavor profile were strongly size-dependent. Large-sized females and males showed only minor divergence in flavor attributes, whereas small-sized females and males exhibited pronounced differences across multiple flavor dimensions. Within the same sex, large-sized females had a relatively simpler flavor composition compared with small-sized females, which harbored a greater diversity and higher relative abundance of flavor compounds; in contrast, no significant size-based difference in flavor substances was observed in male crayfish.

These findings fill a gap in basic flavor chemistry data for farmed red claw crayfish, and have practical applications for size- and sex-selective harvesting, quality grading, and high-quality aquaculture production of *C. quadricarinatus*. For future research, further investigation into the formation mechanisms of key flavor compounds during growth and gonadal development, as well as sensory validation of key volatile profiles, would help support precise flavor regulation and selective breeding of this commercially important crustacean species.

## Figures and Tables

**Table 1:** Muscle composition of experimental animals *C. quadricarinatus.*

	Body Weight (g)	Meat Yield (%)	Crude Protein (%)	Crude Lipid (%)	Moisture (%)	Ash (%)
BF	51.35 ± 5.66 ^c^	16.20 ± 1.41 ^b^	17.76 ± 1.62 ^b^	0.63 ± 0.03 ^b^	80.52 ± 0.76 ^b^	1.28 ± 0.04 ^a^
BM	67.23 ± 14.46 ^b^	17.15 ± 1.29 ^b^	17.12 ± 1.79 ^b^	0.61 ± 0.04 ^b^	81.16 ± 0.98 ^b^	1.32 ± 0.09 ^a^
SF	25.86 ± 4.99 ^a^	18.52 ± 2.37 ^a^	19.71 ± 2.54 ^a^	0.71 ± 0.02 ^a^	78.32 ± 0.54 ^a^	1.38 ± 0.03 ^a^
SM	26.37 ± 4.69 ^a^	17.10 ± 1.75 ^a^	20.06 ± 2.17 ^a^	0.70 ± 0.05 ^a^	78.25 ± 0.69 ^a^	1.41 ± 0.07 ^a^

Values in the same column with different superscript lowercase letters represent a significant difference (*p* < 0.05).

**Table 2:** ROAV values of the important and key odor compounds in the muscle of *C. quadricarinatus.*

Classification	Odor Compound	Representative Odor	ROAV Value			
			MUBF	MUBM	MUSF	MUSM
Alcohols	Ethanol	Sweet	100.00000	100.00000	100.00000	94.20721
	1-Octen-3-ol	Sweet, Fatty, Earthy	63.45633	55.22831	21.32623	100.00000
	1-Hexanol	Sweet, Green, Grassy	3.13797	2.37594	0.96361	4.03241
	1-Heptanol	Sweet, Nutty	2.30467	1.69463	0.50591	2.53211
	Benzyl alcohol	Rose/flower	0.55835	0.43776	0.13876	0.57837
	1-Pentanol	Sweet, Fatty, Grassy	0.47315	0.40231	0.15944	1.08564
	(Z)-5-Octen-1-ol	Grassy, Earthy	0.14990	0.25245	0.07378	0.35420
	1-Penten-3-ol	Fishy, Mushroom, Grassy	0.13355	0.23387	0.08089	0.36679
Aldehydes	2-methyl-Butanal	Balsamic	2.45901	3.20749	1.19183	3.53339
	Nonanal	Fatty, Soapy, Fruity	0.72839	0.98944	0.38546	1.46528
	Benzaldehyde	Nutty, Metallic	0.56812	0.73254	0.30043	1.00458
	Hexanal	Fishy, Grassy	0.74757	0.53532	0.26128	0.73000
	Heptanal	Fishy, Green, Fatty	0.18551	0.19706	0.08648	0.23623
	Octanal	Fatty, Fruity, Grassy	0.10140	0.16906	0.07004	0.12927
	Pentanal	Fatty, Oil	0.11663	0.15187	0.04582	0.24054
	(Z)-4-Heptenal	Fishy	0.23480	0.02199	0.00149	0.06280
Ketones	2,3-Pentanedione	Sweet, Fruity, Nutty	0.25430	0.21862	0.06070	0.28067
	2-Octanone	Fruity, Grassy	0.06785	0.09706	0.03541	0.11362
	2-Heptanone	Fruity	0.04515	0.02819	0.01755	0.10452
Esters	Ethyl Acetate	Sweet, Aromatic, Anise	1.10070	0.62096	0.85605	0.70468
Aromatic hydrocarbons	Styrene	Balsam	0.23756	0.37784	0.17660	0.47572

## Data Availability

The original contributions presented in the study are included in the article/Supplementary Material; further inquiries can be directed to the corresponding authors.

## Supplementary Materials

The following supporting information can be downloaded at: https://www.mdpi.com/article/10.3390/foods15203671/s1. Table S1 the significantly different volatile compound between the MUBF and MUBM groups. Table S2 the significantly different volatile compound between the MUSF and MUSM groups. Table S3 the significantly different volatile compound between the MUBF and MUSF groups. Table S4 the significantly different volatile compound between the MUBM and MUSM groups.
