# Supplementary material for: Flavoromics Analysis Reveals Sex- And Size-Dependent Volatile Flavor Signatures in Cherax quadricarinatus Muscle Under Identical Farming Conditions
Source: Foods. 2026 Oct 15;15(20):3671. doi: 10.3390/foods15203671 (PMC13704413; doi:10.3390/foods15203671)
Supplement: Supplementary file 1 [file foods-15-03671-s001.zip › foods-4572805-supplementary.pdf]

Supplementary Table S1 the significantly different volatile compound between the MUBF and MUBM groups.

		Compound	CAS	Classification	Mean MUBF	Std MUBF	Mean MUBM	Std MUBM	VIP	P-Value	Log_2 (Foldchange)
MUBF vs MUBM	Up	Betazole	105-20-4	Organoheterocyclic compounds	5.98E-07	8.03E-08	8.57E-07	1.68E-07	1.84	0.01434	0.52
		Hydroxylamine, O-(phenylmethyl)-	622-33-3	Amines	4.16E-04	6.25E-04	1.32E-03	3.00E-04	1.61	0.01913	1.67
		unknown50			1.52E-04	1.65E-04	3.97E-04	1.39E-04	1.29	0.03466	1.38
		Methanesulfonyl chloride	124-63-0	Hydrocarbons	2.59E-04	2.55E-04	6.31E-04	2.24E-04	1.28	0.03950	1.29
		Ethanol, 2-butoxy-	111-76-2	Ethers	4.80E-03	4.66E-04	6.23E-03	1.29E-03	1.55	0.04873	0.38
		Silane, tetramethyl-	75-76-3	Others	3.16E-05	4.30E-05	1.03E-04	1.77E-05	1.69	0.00881	1.71
		2-Nonyne	19447-29-1	Hydrocarbons	5.98E-07	8.03E-08	8.57E-07	1.68E-07	1.84	0.01434	0.52
		Furan, 2-butyltetrahydro-	1004-29-1	Organoheterocyclic compounds	1.20E-03	2.81E-04	1.81E-03	4.19E-04	1.74	0.02601	0.60
		unknown177			1.62E-04	1.55E-04	3.68E-04	8.19E-05	1.22	0.03052	1.18
		3-Pyridinol	109-00-2	Organoheterocyclic compounds	5.98E-07	8.03E-08	8.57E-07	1.68E-07	1.84	0.01434	0.52
		2,4-Di-tert-butylphenol	96-76-4	Phenols	1.58E-03	2.17E-04	2.25E-03	6.09E-04	1.58	0.04960	0.51
		1,2-Benzenedicarboxylic acid, diheptyl ester	3648-21-3	Esters	1.82E-05	3.93E-05	1.02E-04	6.79E-05	1.55	0.04345	2.49

	1,2-Benzenedicarboxylic acid, dihexyl ester	84-75-3	Phenols	6.71E-04	9.19E-04	2.57E-03	5.42E-04	1.61	0.00409	1.94
	Methyl 3-hydroxytetradecanoate	55682-83-2	Alcohols	5.98E-07	8.03E-08	8.57E-07	1.68E-07	1.84	0.01434	0.52
	Tetraglyme	143-24-8	Ethers	5.98E-07	8.03E-08	8.57E-07	1.68E-07	1.84	0.01434	0.52
	unknown202			5.98E-07	8.03E-08	8.57E-07	1.68E-07	1.84	0.01434	0.52
Down	Ethyl Acetate	141-78-6	Esters	3.63E-03	1.02E-03	1.99E-03	5.50E-04	1.83	0.01331	-0.87
	3-Heptanone, 4-methyl-	6137-11-7	Ketones	4.45E-04	3.30E-04	8.57E-07	1.68E-07	1.85	0.03942	-9.02
	Acetonitrile	75-05-8	Organic nitrogen compounds	1.50E-02	3.09E-03	9.75E-03	2.34E-03	1.79	0.01690	-0.62
	2-Propanol, 1-methoxy-	107-98-2	Alcohols	1.11E-01	2.64E-02	6.28E-02	3.01E-02	1.44	0.02728	-0.82
	2,2-Dimethyl-3-heptanone	19078-97-8	Others	1.55E-04	3.66E-05	5.69E-05	5.47E-05	1.47	0.01038	-1.44
	2-Hydroxy-3-pentanone	5704-20-1	Ketones	4.37E-04	1.04E-04	1.70E-04	1.26E-04	1.23	0.00662	-1.36
	exo-2-Bromonorbornane			8.61E-05	5.29E-05	8.57E-07	1.68E-07	1.83	0.02267	-6.65
	Pyrrole	109-97-7	Amines	7.51E-04	9.22E-05	4.36E-04	1.19E-04	1.93	0.00157	-0.78
	Hex-2-yn-4-one, 2-methyl-	52066-33-8	Others	1.36E-04	8.33E-05	8.57E-07	1.68E-07	1.84	0.02214	-7.31

Supplementary Table S2 the significantly different volatile compound between the MUSF and MUSM groups.

		Compound	CAS	Classification	Mean MUSF	Std MUSF	Mean MUSM	Std MUSM	VIP	P-Value	Log_2 (Foldchange)
MUSF vs MUSM	Up	Cyclohexane, ethyl-	1678-91-7	Hydrocarbons	4.56E-07	1.38E-07	1.04E-06	2.79E-07	1.69	0.00302	1.19
		Methyl Alcohol	67-56-1	Alcohols	5.10E-03	1.23E-03	1.43E-02	4.42E-03	1.75	0.00794	1.48
		2-Butanone	78-93-3	Ketones	8.58E-03	4.71E-03	1.89E-02	8.64E-03	1.33	0.04780	1.14
		unknown15			4.56E-07	1.38E-07	1.39E-06	7.06E-07	1.61	0.04009	1.61
		Pentanal	110-62-3	Aldehydes	4.21E-04	4.48E-04	1.50E-03	8.03E-04	1.36	0.03052	1.83
		Trichloromethane	67-66-3	Halohydrocarbons	1.25E-03	3.31E-04	2.52E-03	6.28E-04	1.63	0.00400	1.01
		Dimethyl sulfone	67-71-0	Organosulfur compounds	4.88E-05	1.08E-04	2.15E-04	1.16E-04	1.51	0.04819	2.13
		Succinimide	123-56-8	Organoheterocyclic compounds	6.26E-04	9.11E-04	2.78E-03	1.03E-03	1.32	0.00797	2.15
		Undecane	1120-21-4	Hydrocarbons	6.30E-04	2.38E-04	1.28E-03	3.36E-04	1.56	0.00742	1.03
		unknown50			7.15E-05	7.35E-05	3.01E-04	1.06E-04	1.13	0.00408	2.07
		Benzene, 1,3-dimethyl-	108-38-3	Aromatic hydrocarbons	8.33E-04	6.38E-04	1.85E-03	3.65E-04	1.43	0.01483	1.15
		1-Butanol	71-36-3	Alcohols	2.66E-03	1.06E-03	6.22E-03	1.97E-03	1.55	0.00736	1.23
		unknown59			3.37E-04	1.73E-04	6.91E-04	1.31E-04	1.52	0.00645	1.04
		Hexadecane	544-76-3	Hydrocarbons	2.08E-04	9.47E-05	3.92E-04	5.98E-05	1.49	0.00640	0.91
		1,3-bis[(1-Ethylpentyl)oxy]-1,1,3,3-tetramethyldisiloxane		Others	1.96E-03	8.16E-04	3.82E-03	1.06E-03	1.54	0.01444	0.96

unknown72			1.46E-04	4.13E-05	3.40E-04	1.19E-04	1.64	0.00881	1.22
Furan, 2-hexyl-	3777-70-6	Organoheterocyclic compounds	1.11E-04	1.19E-04	5.90E-04	2.31E-04	1.27	0.00332	2.41
3-Buten-1-ol, 3-methyl-	763-32-6	Alcohols	3.23E-04	2.42E-04	1.28E-03	4.58E-04	1.03	0.00320	1.99
Styrene	100-42-5	Aromatic hydrocarbons	4.87E-04	2.08E-04	8.90E-04	2.62E-04	1.38	0.02746	0.87
1-Pentanol	71-41-0	Alcohols	1.47E-02	6.02E-03	6.77E-02	3.04E-02	1.62	0.01629	2.21
Acetoin	513-86-0	Ketones	2.36E-04	1.81E-04	6.79E-04	3.85E-04	1.02	0.04816	1.53
Erythro-3-bromo-2-pentanol	159475-12-4	Alcohols	4.56E-07	1.38E-07	1.04E-06	2.79E-07	1.69	0.00302	1.19
unknown99			7.47E-05	4.41E-05	2.99E-04	6.40E-05	1.11	0.00020	2.00
Nonanal	124-19-6	Aldehydes	2.95E-04	1.57E-04	7.61E-04	3.20E-04	1.50	0.01916	1.37
Ethanol, 2-butoxy-	111-76-2	Ethers	2.95E-03	8.78E-04	6.62E-03	1.67E-03	1.69	0.00247	1.17
exo-2-Bromonorbornane			4.56E-07	1.38E-07	1.04E-06	2.79E-07	1.69	0.00302	1.19
Cyclobutanecarbonitrile	4426-11-3	Organic nitrogen compounds	4.56E-07	1.38E-07	1.04E-06	2.79E-07	1.69	0.00302	1.19
1-Octen-3-ol	3391-86-4	Alcohols	1.63E-02	9.83E-03	5.19E-02	2.63E-02	1.34	0.02192	1.67
unknown116			5.90E-05	3.29E-05	2.02E-04	5.92E-05	1.06	0.00150	1.78
Pyrrole	109-97-7	Amines	2.99E-04	1.27E-04	5.97E-04	2.37E-04	1.26	0.03819	1.00
unknown123			1.50E-03	7.24E-04	2.87E-03	9.37E-04	1.17	0.03232	0.94
2-Furanmethanol, tetrahydro-, acetate	637-64-9	Organoheterocyclic compounds	3.23E-05	4.45E-05	1.81E-04	4.07E-05	1.40	0.00056	2.49
3,5-Diamino-1,2,4-triazole	1455-77-2	Organoheterocyclic compounds	4.56E-07	1.38E-07	1.04E-06	2.79E-07	1.69	0.00302	1.19

		Trimethylsilyl fluoride	420-56-4	Others	4.56E-07	1.38E-07	1.04E-06	2.79E-07	1.69	0.00302	1.19
		Carbamodithioic acid, diethyl-, methyl ester	686-07-7	Acids	4.56E-07	1.38E-07	1.04E-06	2.79E-07	1.69	0.00302	1.19
		Benzamide, N-(2-piperidinylmethyl)-2,5-bis(2,2,2-trifluoroethoxy)-	54143-55-4	Ethers	4.56E-07	1.38E-07	1.04E-06	2.79E-07	1.69	0.00302	1.19
		unknown186			4.56E-07	1.38E-07	1.04E-06	2.79E-07	1.69	0.00302	1.19
		2,4-Di-tert-butylphenol	96-76-4	Phenols	9.84E-04	2.57E-04	2.10E-03	5.15E-04	1.69	0.00254	1.09
		1,3,5-Trioxane	110-88-3	Organoheterocyclic compounds	4.56E-07	1.38E-07	1.04E-06	2.79E-07	1.69	0.00302	1.19
		Ethane, 1,1,1-trimethoxy-	1445-45-0	Esters	4.56E-07	1.38E-07	1.04E-06	2.79E-07	1.69	0.00302	1.19
	Down	Ethyl Acetate	141-78-6	Esters	3.28E-03	8.98E-04	1.83E-03	9.66E-04	1.13	0.03946	-0.84

Supplementary Table S3 the significantly different volatile compound between the MUBF and MUSF groups.

		Compound	CAS	Classification	Mean MUBF	Std MUBF	Mean MUSF	Std MUSF	VIP	P-Value	Log_2 (Foldchange)
MUBF vs MUSF	Down	Methyl Alcohol	67-56-1	Alcohols	8.41E-03	8.22E-04	5.10E-03	1.23E-03	1.87	0.00104	-0.72
		1,3-Dioxolane	646-06-0	Organoheterocyclic compounds	2.06E-03	7.72E-04	9.53E-04	4.34E-04	1.70	0.02318	-1.11
		Acetone	67-64-1	Ketones	2.34E-04	7.00E-05	9.17E-05	7.44E-05	1.08	0.01430	-1.35
		Trichloromethane	67-66-3	Halohydrocarbons	2.84E-03	7.24E-04	1.25E-03	3.31E-04	2.00	0.00210	-1.18
		2,3-Pentanedione	600-14-6	Ketones	3.35E-03	1.47E-03	9.30E-04	9.43E-04	1.11	0.01456	-1.85

Dimethyl sulfone	67-71-0	Organosulfur compounds	3.61E-04	1.90E-04	4.88E-05	1.08E-04	1.83	0.01269	-2.89
Undecane	1120-21-4	Hydrocarbons	9.41E-04	1.77E-04	6.30E-04	2.38E-04	1.47	0.04661	-0.58
1-Butanol	71-36-3	Alcohols	5.02E-03	3.17E-04	2.66E-03	1.06E-03	1.82	0.00581	-0.92
1-Butanol, 2-methyl-	137-32-6	Alcohols	1.92E-03	6.34E-04	9.61E-04	4.77E-04	1.43	0.02663	-1.00
1-Butanol, 3-methyl-	123-51-3	Alcohols	2.27E-03	6.99E-04	1.06E-03	6.34E-04	1.52	0.02118	-1.10
unknown72			2.40E-04	3.38E-05	1.46E-04	4.13E-05	1.80	0.00450	-0.71
2,2-Dimethyl-3-heptanone	19078-97-8	Others	1.55E-04	3.66E-05	1.84E-05	2.45E-05	1.67	0.00012	-3.07
1-Pentanol	71-41-0	Alcohols	3.74E-02	5.27E-03	1.47E-02	6.02E-03	1.79	0.00022	-1.35
Acetoin	513-86-0	Ketones	6.13E-04	1.81E-04	2.36E-04	1.81E-04	1.04	0.01092	-1.38
2-Hydroxy-3-pentanone	5704-20-1	Ketones	4.37E-04	1.04E-04	1.48E-04	1.02E-04	1.09	0.00222	-1.56
1-Hexanol	111-27-3	Alcohols	2.07E-02	2.75E-03	7.38E-03	3.78E-03	1.80	0.00022	-1.49
Nonanal	124-19-6	Aldehydes	4.80E-04	5.14E-05	2.95E-04	1.57E-04	1.49	0.03660	-0.70
Ethanol, 2-butoxy-	111-76-2	Ethers	4.80E-03	4.66E-04	2.95E-03	8.78E-04	1.78	0.00314	-0.70
unknown108			8.25E-05	5.32E-05	4.56E-07	1.38E-07	1.93	0.02606	-7.50
exo-2-Bromonorbornane			8.61E-05	5.29E-05	4.56E-07	1.38E-07	1.70	0.02233	-7.56
1-Octen-3-ol	3391-86-4	Alcohols	4.18E-02	7.99E-03	1.63E-02	9.83E-03	1.61	0.00200	-1.36
1-Heptanol	111-70-6	Alcohols	4.56E-03	1.57E-03	1.16E-03	7.64E-04	1.08	0.00245	-1.97
Pyrrole	109-97-7	Amines	7.51E-04	9.22E-05	2.99E-04	1.27E-04	1.88	0.00020	-1.33

	unknown123			2.60E-03	5.89E-04	1.50E-03	7.24E-04	1.53	0.02932	-0.80
	Hex-2-yn-4-one, 2-methyl-	52066-33-8	Others	1.36E-04	8.33E-05	2.41E-05	5.29E-05	1.48	0.03475	-2.50
	1,3-Benzenediol, monobenzoate	136-36-7	Phenols	1.99E-04	2.01E-05	8.46E-05	5.69E-05	1.03	0.00278	-1.24
	Hexanoic acid, anhydride	2051-49-2	Acids	3.36E-04	1.68E-04	1.01E-04	7.87E-05	1.13	0.02172	-1.74
	Benzyl alcohol	100-51-6	Alcohols	4.42E-04	1.71E-04	1.28E-04	1.37E-04	1.33	0.01252	-1.79
	Furan, 2-butyltetrahydro-	1004-29-1	Organoheterocyclic compounds	1.20E-03	2.81E-04	4.97E-04	4.23E-04	1.10	0.01497	-1.27
	2,4-Di-tert-butylphenol	96-76-4	Phenols	1.58E-03	2.17E-04	9.84E-04	2.57E-04	1.77	0.00414	-0.68

Supplementary Table S4 the significantly different volatile compound between the MUBM and MUSM groups.

		Compound	CAS	Classification	Mean MUBM	Std MUBM	Mean MUSM	Std MUSM	VIP	P-Value	Log_2 (Foldchange)
MUBM vs MUSM	Up	2-Heptanone	110-43-0	Ketones	3.61E-04	3.61E-04	1.09E-03	5.76E-04	1.52	0.04429	1.59
	Down	1-Octanol	111-87-5	Alcohols	9.21E-04	6.79E-04	1.04E-06	2.79E-07	2.16	0.03884	-9.79
		1,2-Benzenedicarboxylic acid, dihexyl ester	84-75-3	Phenols	2.57E-03	5.42E-04	4.47E-04	9.98E-04	2.29	0.00310	-2.52
